# Effect of a Low-Calorie Dietary Intervention on Liver Health and Body Weight in Adults with Metabolic-Dysfunction Associated Steatotic Liver Disease (MASLD) and Overweight/Obesity: A Systematic Review and Meta-Analysis

**DOI:** 10.3390/nu16071030

**Published:** 2024-04-01

**Authors:** Laurence J. Dobbie, Jamie Burgess, Azlinda Hamid, Sarah J. Nevitt, Theresa J. Hydes, Uazman Alam, Daniel J. Cuthbertson

**Affiliations:** 1 Department of Cardiovascular and Metabolic Medicine, Institute of Life Course and Medical Sciences, University of Liverpool, Liverpool L9 7AL, UK; laurence.dobbie@kcl.ac.uk (L.J.D.);; 2 Department of Diabetes & Endocrinology, Guys Hospital, Guy’s and St Thomas’ NHS Foundation Trust, London SE1 9RT, UK; 3 University Hospital Aintree, Liverpool University Hospitals NHS Foundation Trust, Liverpool L9 7AL, UK; 4 Department of Health Data Science, Institute of Population Health, University of Liverpool, Liverpool L3 5TR, UK; sarah.nevitt@york.ac.uk; 5 Centre for Reviews and Dissemination, University of York, York YO10 5DD, UK

**Keywords:** overweight, obesity, total-diet replacement, Very-Low-Calorie Diet, MASLD, randomised controlled trial, lifestyle medicine

## Abstract

Introduction: Metabolic-dysfunction Associated Steatotic Liver Disease (MASLD) is a common cause of chronic liver disease. This review assessed the efficacy of a Low-Calorie Diet (LCD) on liver health and body weight in people living with MASLD and obesity. Methods: The study was registered with PROSPERO (CRD42021296501), and a literature search was conducted using multiple databases. The key inclusion criteria were randomised controlled trials or cohort studies, obesity/overweight and MASLD. Two authors screened abstracts, reviewed full texts and performed data extraction and quality assessment. The primary outcome was the change in the serum ALT, and secondary outcomes included the changes in the serum AST, intrahepatic lipid content (IHL), quantified non-invasively via MRI/MRS, and body weight. Results: Fifteen studies were included. The LCD reduced body weight by 9.1 kg versus the control (95%CI: −12.4, −5.8) but not serum ALT (−5.9 IU/L, −13.9, 2.0). Total Dietary Replacement (TDR) reduced IHL by −9.1% vs. the control (−15.6%, −2.6%). The Mediterranean-LCD for ≥12 months reduced ALT (−4.1 IU/L, −7.6, −0.5) and for 24 months reduced liver stiffness versus other LCDs. The Green-Mediterranean-LCD reduced IHL, independent of body weight. Limited studies assessed those of Black or Asian ethnicity, and there was heterogeneity in the methods assessing the liver fat content and fibrosis. Conclusions: In people with MASLD and obesity, an LCD intervention reduces IHL and body weight. Trials should focus on the recruitment of Black and Asian ethnicity participants.

## 1. Introduction

Non-alcoholic Fatty Liver Disease (NAFLD), recently renamed metabolic-dysfunction associated steatotic liver disease (MASLD), is a highly prevalent obesity-related liver disease. MASLD is often denoted as the ‘hepatic manifestation of the metabolic syndrome’ [1,2], with the disease spectrum ranging from hepatic steatosis (fatty liver) to metabolic-associated steatohepatitis (MASH) and cirrhosis [3]. Lifestyle intervention, including diet and physical activity, is an effective intervention for MASLD [4]. Optimal benefits are achieved using a combination of weight loss (aiming for at least 7% weight loss) and increased physical activity [5,6,7,8]. Dietary interventions, including the low-calorie diet (LCD), have been investigated for their effect on intrahepatic lipid and associated liver fibro-inflammation. 

The LCD is defined as either a total intake of 800–1500 kcal/day, a 500 kcal/day calorie reduction or a 25% calorie reduction [9,10,11]. The LCD has demonstrated efficacy in reducing body weight and inducing the remission of type 2 diabetes (T2D). In the DIRECT trial of participants with T2D diagnosed within the last 6 years, a total-diet replacement (TDR) intervention (823–853 kcal/day) for 3–5 months led to significant body weight reduction and a T2D remission rate of 46% after 12 months, dependent on the weight loss magnitude [12]. The mechanism of action related to reductions in ectopic liver and pancreatic fat improves insulin sensitivity and insulin secretion [13]. Similarly, the DROPLET study showed that TDR in people with obesity reduced body weight by >10% in 45% of participants [14]. Importantly, there are dose-dependent associations between weight reduction and reduction in hepatic steatosis; therefore, at the level of weight loss achieved with TDR, there may be a benefit for MASLD and steatosis reversal [15]. Furthermore, the MASLD prevalence in T2D is 65% [16], with insulin resistance and inflammation contributing towards the hepatic triglyceride accumulation that characterises MASLD [17]. Therefore, the LCD, particularly TDR, may be an effective approach for MASLD via reductions in body weight and improving insulin resistance and glycaemic control.

The Mediterranean diet is characterised by a minimal consumption of processed/ultra-processed foods and a high consumption of whole grains, nuts, legumes, olive oil, vegetables and fruits [18]. Population-level data indicate that adherence to a Mediterranean diet reduces MASLD prevalence [19]. Similarly, the ATTICA observational study showed an inverse relationship between the Mediterranean diet and MASLD [20]. Polyphenols, a potent plant antioxidant and a component of the ‘Green-Mediterranean diet’, may also be of benefit. Polyphenols are abundant in green tea and walnuts and reduce de novo lipogenesis, inflammation and fatty acid beta-oxidation; this could benefit ectopic liver fat [21,22]. The combination of a Mediterranean diet, a high polyphenol content and calorie restriction requires evaluation as an MASLD therapy. 

Although EASL-EASD-EASO, ESPEN and APASL guidelines [5,6,7] advocate for weight loss of 7–10% where possible for patients with MASLD, the most effective way to achieve this has not been well described. Potential therapeutic strategies could include the LCD, TDR or a calorie-restricted Mediterranean diet with or without added dietary polyphenols. This systematic review aims to determine the impact of a Low-Calorie Diet on liver health and body weight versus a control intervention in people living with MASLD and obesity. We also aimed to determine the efficacy of consuming various types of LCD including TDR, Mediterranean-LCD and Green-Mediterranean-LCD.

## 2. Materials and Methods

### 2.1. Search Strategy 

This study protocol was registered a priori with The International Prospective Register of Systematic Reviews (PROSPERO) (Registration Number: CRD42021296501). The search strategy was developed by a medical librarian at Liverpool University Hospitals National Health Service (NHS) Foundation Trust. Relevant studies were identified by systematically searching MEDLINE, PubMed, EMBASE and Cumulative Index to Nursing and Allied Health Literature (CINAHL) (Supplementary Methods S1). All articles published up until 31 December 2021 were included, with articles restricted to the English language. In addition, a PubMed search up until 30 November 2022 was repeated before the evidence synthesis. The initial database search was conducted by two authors (LD, JB). Review articles were screened for references, and the authors were contacted to provide relevant studies for assessment. All relevant articles from each database were merged using Rayyan, and duplicates were excluded. Our PROSPERO registration included a research question to compare LCD against metabolic/bariatric surgery. No studies examining bariatric surgery against LCD were highlighted, so this research question was not addressed.

### 2.2. Definition of Low-Calorie Diet and Very-Low-Calorie Diet

We defined LCD and VLCD based on previously published definitions (Table 1) [9,10,11].

### 2.3. Systematic Review Aim

The systematic review aims were set a priori. This review aimed to determine the efficacy of the LCD in the treatment of people with MASLD and obesity versus the control, conventional care, Very-Low-Calorie Diet (VLCD), other dietary interventions and bariatric/metabolic surgery.

### 2.4. Inclusion/Exclusion Criteria

Table 2 details the inclusion and exclusion criteria for the selection of studies for this systematic review. 

### 2.5. Outcome Measures

The primary outcome measure was the mean change in the serum alanine transaminase (ALT) concentration between the intervention and comparator groups.

Secondary outcome measures included:

1. **Biochemical outcomes:** Change in Aspartate Aminotransferase (AST), Gamma-Glutamyl Transpeptidase (GGT), Fatty Liver Index (FLI) and SteatoTest.
2. **Body anthropometry outcomes:** Change in body weight, body mass index (BMI) and body fat percentage.
3. **Liver imaging outcomes:** Ultrasound (echogenicity), Transient elastography (including measurement of controlled attenuation parameter (CAP)), MRI-determined measurements of liver fat (PDFF, proton density fat fraction), Magnetic resonance spectroscopy (hepatic fat fraction) and Magnetic resonance elastography.
4. **Liver histology:** Regression of histological changes in MASLD on liver biopsy, improvement in steatosis/reduction in NASH Activity Score (NAS) and improvement in fibrosis.

### 2.6. Literature Screening

Two authors (L.D. and J.B.) independently screened abstracts using Rayyan. If there was any doubt about the inclusion of an article, this was included for full-text review. Full articles were screened independently by two authors (L.J.D. and J.B.) using Rayyan. Disagreements were resolved by returning to the original article along with a third senior author (D.J.C.), when required.

### 2.7. Data Extraction

Data were extracted from each study independently by one author (L.J.D.) using a standardised data extraction sheet on REDCAP. The extracted studies were then validated by another author (J.B.) to ensure data integrity. The extracted data were then uploaded into Microsoft Excel and used to perform a narrative synthesis. The extracted data from all studies included general information (title, authors, DOI, journal, year of publication, RCT name), clinical details (percentage with MASLD, percentage with metabolic syndrome, percentage with T2D, age, sex distribution), intervention details (dietary composition, nature of control intervention, duration of intervention), liver outcomes and anthropometric outcomes. 

In the event of missing data, the researchers attempted to contact study investigators for unreported data or additional details. The contact information for the study authors was identified from PubMed or the Internet, and corresponding authors were contacted by email to ask if they could share their study data. 

### 2.8. Quality Assessment

One investigator (L.J.D.) screened all of the included studies for the risk of bias, with a separate investigator (J.B.) independently validating the risk of bias. The Cochrane risk of bias tool was used for randomised controlled trials, and the ROBINS-I (risk of bias in non-randomised studies of interventions) tool was used for cohort studies [23,24]. Disagreements between the investigators reviewing the papers were resolved by discussion and the involvement of a third senior author (D.J.C.), where necessary.

### 2.9. Statistics

All statistics were conducted using R studio (Version: 2022.12.0+353). The R package ‘meta’ was used for the analysis. All data units for glucose and HbA1c were converted to mmol/L or mmol/mol. When calculating summary data for demographic characteristics, if a percentage was reported, this was inputted to the total sample size to calculate accurate numbers. In the case of non-whole numbers, these were rounded to the nearest whole number. Meta-analysis statistics were conducted based on the mean difference between baseline and final values alongside standard deviations. If not reported, the mean change was calculated by subtracting the final mean from the baseline mean. If not reported, standard deviations of change were calculated, as denoted in the Cochrane Handbook [25]. This involved calculating a correlation coefficient to determine the final standard deviation for change. The following mathematical Equations (1) and (2), shown below, were used for the analysis. The correlation coefficients for ALT, body weight and AST were calculated from data from the FLIPAN study [26]. The correlation coefficients were as follows: 0.5108 for ALT, 0.5369 for AST and 0.9252 for body weight. Based on the correlation, the standard deviation of change was then calculated [25].

Mathematical Equation (1):

(1) $$Corr=\frac{{SD}_{E,baseline}^{2}+{SD}_{E,final}^{2}-{SD}_{E,change}^{2}}{2\times {SD}_{E,baseline}\times {SD}_{E,final}}$$

Mathematical Equation (2):

(2) $${SD}_{E,change}=\sqrt{{SD}_{E,baseline}^{2}+{SD}_{E,final}^{2}-(2\times Corr\;x\;{SD}_{E,baseline}\times {SD}_{E,final})}$$

A random-effects model was used for all analyses due to significant heterogeneity in dietary interventions. Statistical heterogeneity was investigated using the *I*^2^ statistics. An *I*^2^ > 0.5 alongside a *p* ≤ 0.05 was taken as indicating heterogeneity. Publication bias was examined using funnel plots. The outcome of the meta-analysis was presented graphically using forest plots. Data for IHL from the DIRECT Trial were re-analysed using the individual raw data presented in the Supplementary Material of the published manuscript to determine mean differences and standard deviations of change [13]. The FLIPAN study and DIRECT-Plus were meta-analysed by merging the two MED diet groups [22,26,27]. For the meta-analysis of Mediterranean-LCD vs. other LCDs, a pre-specified sensitivity analysis was performed to determine the impact of short-term (3–6 months) and long-term intervention (≥12 months) durations.

## 3. Results

### 3.1. Systematic Review and Intervention Characteristics

#### 3.1.1. Included Studies Subsection

**Figure 1:**
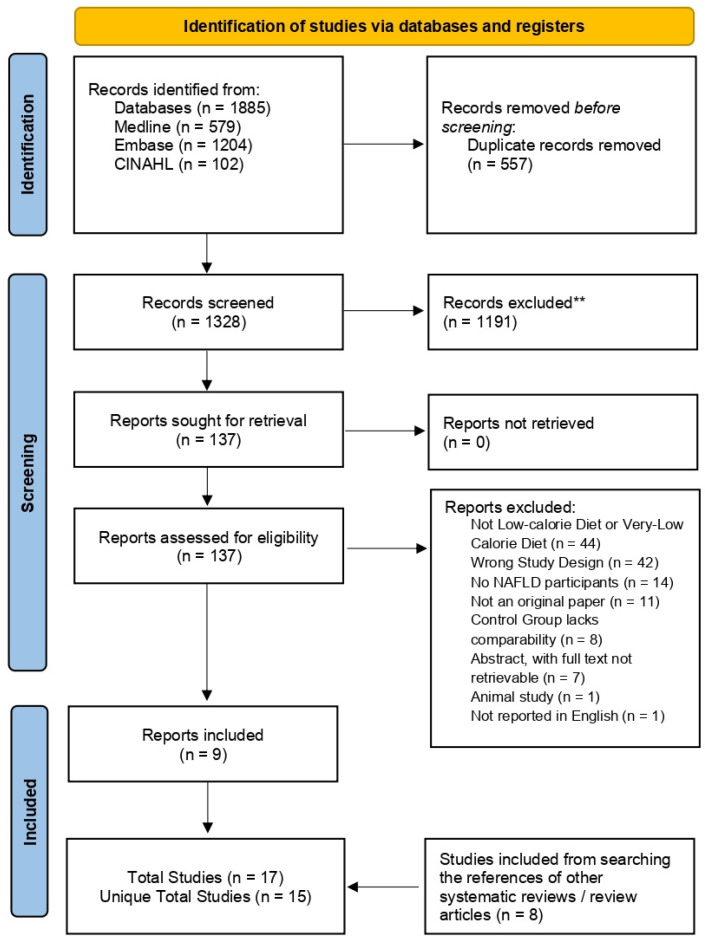
PRISMA Flow Diagram. Figure showing the study selection from the abstract screening to the final study selection. n, number; NAFLD, Non-Alcoholic Fatty Liver Disease.

The study selection process is described in the PRISMA flow chart (Figure 1). Of the 1328 records identified, 9 full texts were included. From searching the references of other reviews, we identified a further 8 studies. Two full texts were from a study already included, bringing the final sample to 15 studies (13 RCTs, 1 cohort study and 1 clinical trial, which was only semi-randomised) [12,13,22,26,27,28,29,30,31,32,33,34,35,36,37,38,39,40]. Nine studies were included in the meta-analysis [12,13,22,26,27,30,31,34,36,39,40,41,42].

The characteristics of the included trials are detailed in Table 3 and Supplementary Tables S1 and S2. The included studies were published between 2010 and 2022 and included a total of 1314 participants. The studies were conducted in Spain (three studies, *n* = 270), Israel (two studies, *n* = 572), USA (two studies, *n* = 49), Brazil (two studies, *n* = 77), the United Kingdom (two studies, *n* = 115), China (one study, *n* = 44), Serbia (one study, *n* = 27), Saudi Arabia (one study, *n* = 100) and Iran (one study, *n* = 60). In terms of the location of trials, 59.1% were in Asia (*n* = 776), 31.4% were in Europe (*n* = 412), 5.86% were in South America (*n* = 77), 3.7% were in North America (*n* = 49) and no trials were in Africa or Oceania. The sample size of the included studies ranged from 18 to 294 participants per trial, and 70.7% were male (*n* = 929). The participants across all trials had a mean age of 48.5 ± 9.2 years. The duration of the interventions ranged from 2 weeks to 18 months. The T2D prevalence ranged from 0% to 100% and was not stated in four studies. The mean BMI for all studies included was in keeping with obesity. The MASLD prevalence ranged from 53% to 100%.

#### 3.1.2. Intervention Characteristics

Seven trials assessed LCD vs. control [12,13,36,37,38,39,40,41]. Two of the LCD trials assessed TDR vs. control in people with recently diagnosed T2D [12,13,40]. Four trials assessed Mediterranean-LCD vs. LCD [26,28,29,30,31,34]. One Trial assessed the green-Mediterranean-LCD vs. Mediterranean-LCD vs. control [22,27]. Further studies assessed LCD vs. low-carbohydrate diet [32], Very-Low-Calorie ketogenic diet vs. LCD [33], Mediterranean-LCD vs. low-fat diet and LCD-monounsaturated fatty acids vs. LCD-polyunsaturated fatty acids [35]. Dietary components within interventions all included an energy prescription ranging from 600 to 1800 kcal/day. 

#### 3.1.3. Studies Nearly Meeting Inclusion

Nine studies nearly met inclusion [14,43,44,45,46,47,48,49,50]. These are detailed in Supplementary Table S3. These were excluded for the following reasons: four did not include MASLD participants [47,48,49,50], two did not have a control group [44,46], two did not define MASLD prevalence [14,45] and one did not meet the calorie reduction definition for LCD [43]. The DROPLET study was not included, as it did not define MASLD prevalence at baseline [14].

### 3.2. Risk of Bias Assessment

Thirteen randomised controlled trials were quality-assessed using the Cochrane risk of bias tool (Supplementary Figure S1A). Seven trials were at a low risk of bias [12,13,22,26,27,28,29,34,40,41], four had some concerns [30,31,33,35,39] and two were at a high risk of bias [36,38]. Two non-randomised controlled trial studies were assessed using the ROBINS-I tool (Supplementary Figure S1B): one study was at a serious risk of bias [32] and one was at a critical risk of bias [37].

### 3.3. Trials Assessing LCD vs. Control (Including TDR)

#### 3.3.1. Trial Demographics

Six RCTs and one cohort study assessed the LCD versus control [12,13,36,37,38,39,40,41]. These trials included 381 participants and were conducted in the UK (two studies), Iran (one study), USA (one study), Saudi Arabia (one study), Brazil (one study) and China (one study). The sample size for the included studies ranged between 25 and 100, 62.7% were male (*n* = 239) and the mean age was 47.3 ± 8.0 years. The intervention duration ranged from 2 to 12 months. 

#### 3.3.2. Primary Outcome—ALT

**Figure 2:**
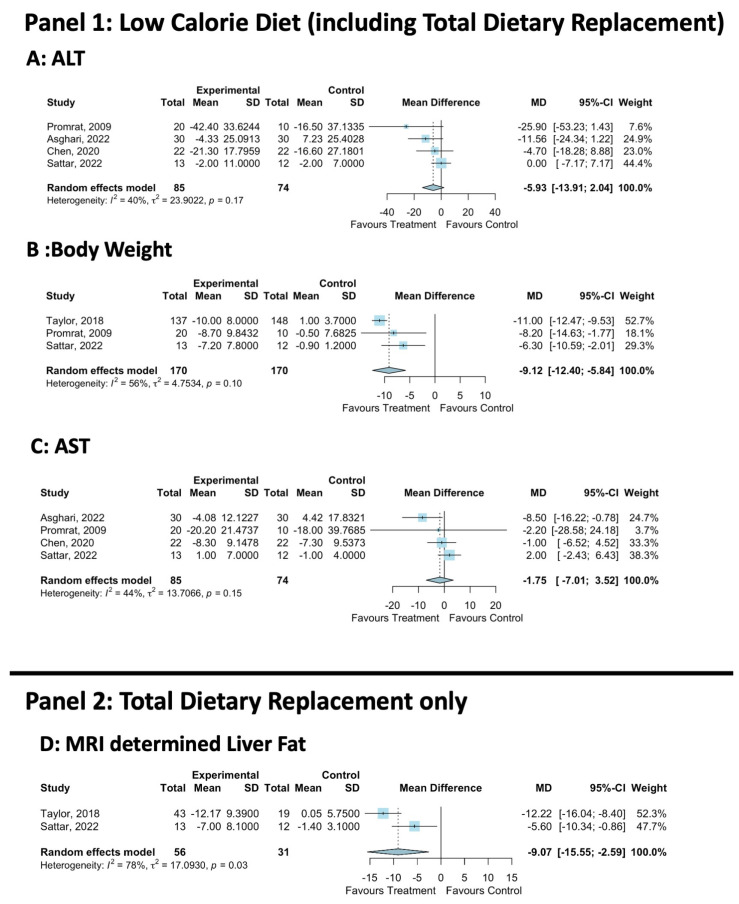
Meta-analysis of LCD vs. control effect on body weight and liver health. MD, Mean Difference; SD, Standard Deviation; ALT, Alanine Transaminase; AST, Aspartate Aminotransferase [12,13,36,39,40,41].

ALT was reported in six trials [36,37,38,39,40,41]. The meta-analysis of ALT included four trials [36,39,40,41] and showed a non-significant mean difference of −5.9 IU/L between the LCD and the control (MD: −5.9, 95% CI: −13.9, 2.0, LCD = 85, Control = 74, Figure 2A). There was a low level of statistical heterogeneity (*I*^2^ = 40%, *p* = 0.17), and the funnel plot did not demonstrate publication bias (Supplementary Figure S2).

#### 3.3.3. Secondary Liver Health Outcomes

Six trials reported AST [36,37,38,39,40,41]. The meta-analysis showed that the LCD did not significantly reduce AST (LCD = 85, Control = 74, 4 studies, MD: −1.8, 95%CI: −7.0, 3.5, Figure 2C). There was a low level of statistical heterogeneity (*I*^2^: 44%, *p* = 0.15), and the funnel plot did not demonstrate publication bias (Supplementary Figure S3). IHL was analysed in four trials via MR-spectroscopy [12,13,40], CT scan liver density [37] or liver ultrasound [41]. All studies showed that the LCD significantly reduced IHL vs. the control. Liver histology was reported in one trial [39], which demonstrated that the NAFLD Activity Score (NAS) was significantly reduced with LCD vs. the control. This was driven by a reduction in fat but not by parenchymal inflammation, ballooning injury or fibrosis [39].

#### 3.3.4. Secondary Body Composition Outcomes

Across all studies, the LCD significantly reduced the body weight and BMI vs. the control [12,13,37,38,39]. The meta-analysis demonstrated that the LCD significantly reduced body weight by −9.1 kg vs. the control (three studies, LCD = 170, Control = 170, MD: −9.1, 95% CI: −12.4, −5.8, Figure 2B). Moderate non-significant statistical heterogeneity was present (*I*^2^ = 56%, *p* = 0.10), and the funnel plot did not demonstrate publication bias (Supplementary Figure S4).

### 3.4. Trials Assessing TDR vs. Control

#### 3.4.1. Trial Demographics

TDR was interrogated against a control arm in two studies which included patients with T2D diagnosed within the last 6 years [12,13,40]. These studies were included, as they included participants based on IHL > 5%, in keeping with steatotic liver disease. Both studies assessed the Counterweight Plus™ weight management programme, which involves TDR followed by a food reintroduction phase, with physical activity. Control participants underwent usual diabetes management via their GP. The duration of the intervention analysis was 3–12 months. STANDBY recruited participants of South Asian ethnicity, whereas DIRECT recruited participants of White European ethnicity. Of note, STANDBY included an initial RCT design of TDR vs. control, with the control group undergoing delayed TDR after 3 months of being on the control arm. This meant the observational analysis could be performed on participants from both groups.

#### 3.4.2. Liver Fat Quantification

The meta-analysis showed that IHL was reduced by −9.1% with TDR vs. the control (LCD = 56, Control = 31, MD: −9.1, 95%CI −15.6, −2.6, Figure 2D). In DIRECT, IHL reduced from 16.0 ± 1.3 to 3.1 ± 0.5 at 5 months and 4.1 ± 0.8 at 12 months; this is in keeping with MASLD remission. In STANDY, this reduced from 16.1 ± 9.0 to 7.0 ± 8.1, which did not meet the threshold for MASLD remission on average. 

#### 3.4.3. Body Composition

In DIRECT, body weight was significantly reduced by −15.3 kg at 5 months and −12.7 kg at 12 months. STANDBY reported that this was −7.2 ± 7.8 kg with immediate TDR and with a between-group difference of −6.3 kg. 

### 3.5. Trials Assessing Mediterranean-LCD vs. Other LCDs

#### 3.5.1. Trial Demographics

Four trials assessed the Mediterranean-LCD vs. other LCDs [26,28,29,30,31,34]. A total of 531 participants were included in trials assessing the Mediterranean-LCD vs. all other LCDs, with two studies conducted in Spain [26,30,31], one in Israel [28,29] and one in Serbia [34]. Of these participants, 76.5% were male (*n* = 406) and 23.5% were female (*n* = 125), the mean age was 48.8 ± 8.6 years and the intervention duration ranged from 3 months to 24 months.

#### 3.5.2. Primary Outcome—ALT

**Figure 3:**
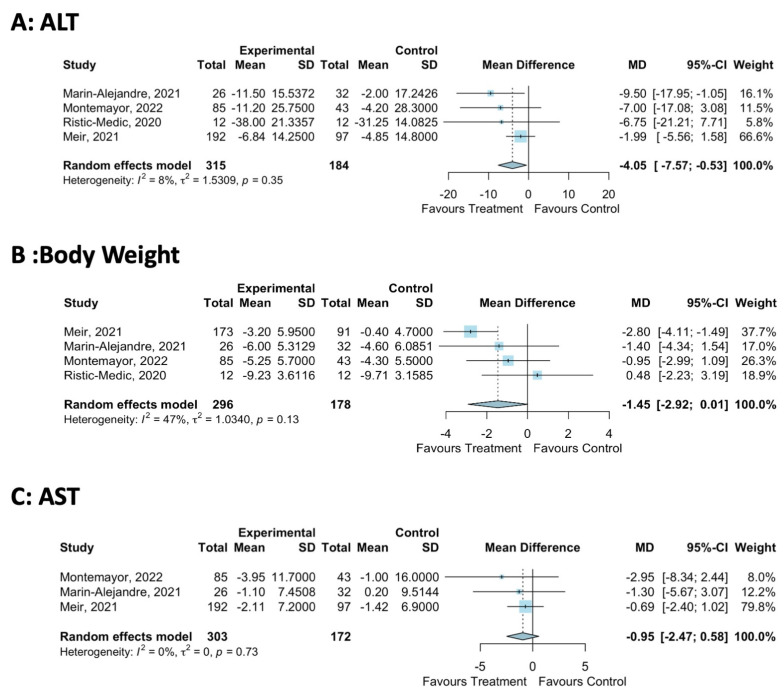
Meta-analysis of Mediterranean-LCD vs. Other LCD/Lifestyle Interventions [22,26,31,34].

All studies assessed the primary outcome of ALT. Only Mediterranean-LCD interventions of 12–24 months significantly reduced ALT [26,31], with shorter 3–6 month interventions not impacting ALT [30,34]. The meta-analysis showed that the Mediterranean-LCD reduced ALT by −4.1 IU/L compared to the other LCD (four studies, MD −4.1 IU/L, 95%CI: −7.6, −0.5, Figure 3A). There was a low level of statistical heterogeneity reported (*I*^2^ = 8%, *p* = 0.35), and the funnel plot did not demonstrate publication bias (Supplementary Figure S6).

#### 3.5.3. Secondary Liver Health Outcomes

Data for AST were reported in four studies [26,30,31,34]. The meta-analysis showed that the Mediterranean-LCD did not significantly reduce AST (three studies, MD: −1.0, 95%CI: −2.5, 0.6, Figure 3C). There was a low level of statistical heterogeneity (*I*^2^: 0%, *p* = 0.73), and the funnel plot did not demonstrate the presence of publication bias (Supplementary Figure S7). Liver Fat data showed that the shorter-duration trials of the Mediterranean-LCD did not significantly reduce IHL compared to other LCDs [26,30]. Longer-duration interventions (12–24 months) showed that the Mediteranean-LCD reduced IHL vs. LCD in some cases [26,28]. For Liver Fibrosis, two trials performed transient elastography. Twenty-four months of the Mediteranean-LCD significantly reduced the liver stiffness measurement determined via transient elastography from baseline (MED-LCD: Baseline 4.7 ± 2 kpa, 24 months: 3.7 ± 1 kpa, *p* < 0.05; American Heart Association Diet Baseline: 5.2 ± 2 kpa, 24 months: 4.8 ± 2, *p* > 0.05, between-group difference for change *p* = 0.016) [31], whereas 6–12 months of the Mediteranean-LCD did not lead to significant improvements in kpa vs. other groups in both the FLIPAN and FLIO trials [26].

#### 3.5.4. Secondary Body Composition Outcomes

Trials showed that the Mediterranean-LCD did not significantly improve measures of body composition compared to other LCDs. The meta-analysis reported that the Mediterranean-LCD trended towards reducing body weight by −1.5 kg vs. other LCD (four studies, MD: −1.5, 95% CI: −2.9, 0.0, Figure 3B). There was a low level of statistical heterogeneity (*I*^2^: 47%, *p* = 0.13), and the funnel plot did not demonstrate publication bias (Supplementary Figure S5)

#### 3.5.5. Sensitivity Analysis of the Effect of the Duration of the Mediterranean-LCD Intervention

**Figure 4:**
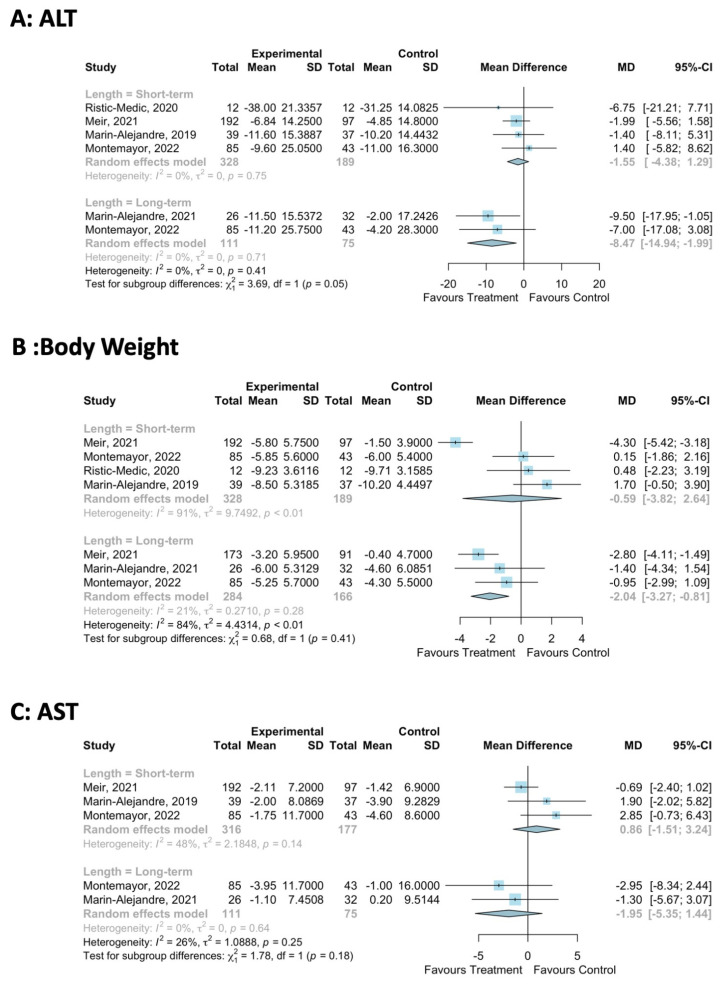
Sensitivity meta-analysis of Mediterranean-LCD vs. other LCD/lifestyle interventions assessing the impact of the duration of the intervention on body weight and liver outcome measures [22,26,30,31,34].

A sensitivity analysis was conducted on the impact of the duration of the Mediterranean-LCD intervention on ALT, AST and body weight. For body weight, 3–6 months of a Mediteranean-LCD did not impact body weight (MD: -0.6, 95%CI: −3.8, 2.6, Figure 4), but 12–24 months significantly reduced body weight by −2.0 kg (MD: −2.0 kg, 95% CI: −3.3, −0.8, Figure 4). For ALT, 3–6 months of Mediteranean-LCD did not impact ALT (MD: −1.6, 95%CI: −4.4, 1.3, Figure 4), but 12–24 months did significantly reduce ALT by −8.5 IU/L (MD: −8.5, 95%CI: −14.9, −2.00, Figure 4). For AST, all durations of the Mediteranean-LCD did not impact AST (Figure 4).

### 3.6. Trials Assessing Green-Mediterranean LCD vs. Mediterranean-LCD and Healthy Dietary Intervention

The DIRECT-PLUS was the only randomised controlled trial to assess the Green-Mediteranean-LCD [22,27]. This 18-month trial took place in Israel and randomised participants into three groups: the healthy dietary group, Meditereanean-LCD and Green-Mediterranean- LCD. There were 294 participants; 88.1% of these were male, 62% were with MASLD, 10.9% were with T2D and 58.8% were with obesity [22,27].

#### 3.6.1. Liver Health

Liver biochemistry 18-month data showed that the green-Mediterranean-LCD reduced ALT and AST vs. the other groups [22]. In comparison, 6 months of green-Mediterranean LCD did not improve ALT or AST vs. the other groups [27]. Absolute numbers were not reported, limiting the analysis. 

Liver fat quantification: The green-Mediterranean-LCD significantly reduced IHL compared to the other groups (relative difference from baseline IHL: healthy dietary group: −12.2%, Mediterranean-LCD: −19.6%, green-Mediterranean-LCD: −38.9%, between groups *p* = 0.023). The MASLD prevalence was 62% at baseline; following intervention, this reduced significantly more in the green-Mediterranean-LCD group (post-intervention MASLD prevalence; healthy dietary group: 54.8%, Mediterranean-LCD: 47.9%, green-Mediterranean-LCD: 31.5%, *p* = 0.012). The differences in IHL between the two Mediterranean dietary groups remained significant after adjustment for 18-month body weight loss (*p* = 0.035) and after adding physical activity and energy intake to the analysis (*p* = 0.047).

#### 3.6.2. Body Composition

**Figure 5:**
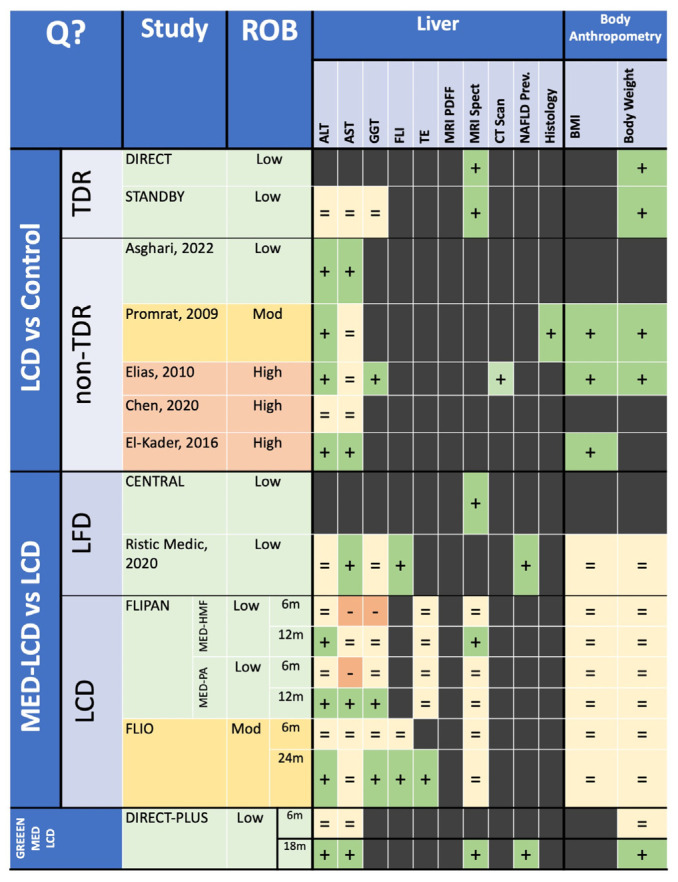
Heatmap of systematic review findings for all interventions. In the study/ROB column, green indicates a low ROB, yellow indicates a moderate ROB and red indicates a high ROB. In the Liver and Body Composition Columns, green with a plus sign indicates a beneficial effect, light-yellow with an equals sign indicates no effect and red with a negative sign indicates a detrimental effect. Q?: Question; ROB: Risk of Bias; ALT: Alanine Transaminase; AST: Aspartate transaminase; GGT: Gamma-Glutamyl Transpeptidase; FLI: Fatty Liver Index; TE: Transient Elastography; BMI: Body Mass Index; LCD: Low-Calorie Diet; TDR: Total-Dietary Replacement; LFD: Low-Fat Diet; MED-LCD: Mediterranean Low-Calorie Diet; GREEN-MED-LCD: Green-Mediterranean Low-Calorie Diet; MED-PA: Mediterranean Diet with Physical Activity; MED-HMF: Mediterranean Diet with High Meal Frequency [34,36,37,38,39,41].

The body weight reduction tended to be higher in the green-Mediterranean-LCD group (mean difference green-Mediterranean-LCD vs. healthy dietary group: −3.2 kg, mean difference Mediterranean-LCD vs. healthy dietary group: −2.3 kg) (Figure 5).

## 4. Discussion

We conducted a systematic review and meta-analysis of RCTs and cohort studies to delineate how the LCD impacts liver health (assessed by the liver enzyme, the quantification of liver fat via imaging, transient elastography and histology) and body anthropometry in people with MASLD and obesity. The results for the primary outcome, ALT, showed that the LCD trended towards reducing ALT vs. the control. The Mediterranean-LCD for >=12 months reduced ALT levels compared to other LCDs despite comparable levels of weight loss. For liver fat, total dietary replacement reduced IHL in those with T2D, MASLD and obesity, with the Mediterranean-LCD improving IHL in some studies. Twenty-four months of the Mediterranean-LCD were also shown to significantly improve liver fibrosis (as measured via transient elastography) vs. other LCDs in one study. The Green-Mediterranean-LCD reduced IHL and liver enzymes, independent of weight loss. Further evaluation of the green-Mediterranean-LCD is required, as this finding is based on only one well-conducted RCT. 

### 4.1. Total Dietary Replacement

We report that, in people with T2D diagnosed in the last 6 years, MASLD and obesity, TDR significantly reduced IHL. However, it has yet to be determined if TDR leads to MASLD remission or is of benefit in those without T2D or with a longer T2D duration. TDR has well-established efficacy in people with T2D and obesity [12,14,50]. In the DIRECT study, TDR normalised hepatic insulin sensitivity and reduced IHL [13]. Importantly, as the duration of T2D increased, the chance of the restoration of beta-cell function reduced [13]. TDR non-responders, who were defined as those not returning to non-diabetic glucose control after weight loss, also had lower baseline IHL levels, lower serum ALT and lower fasting insulin concentrations [13]. Therefore, akin to the DIRECT trials, the potential improvements in MASLD with TDR may be more likely with recently diagnosed MASLD. Further evaluation is required to determine the impact of TDR on liver fibrosis. 

### 4.2. Mediterranean-LCD

Our review highlights that, in people with MASLD and obesity, consuming a Mediterranean-LCD for 12 months or more leads to fewer liver enzymes compared to other LCDs, although changes in body weight were similar and conflicting results were identified for improvement in the IHL content. The Mediterranean diet has well-established health benefits [51]. The CENTRAL trial reported that the Mediterranean-LCD is superior to a low-fat diet in reducing IHL, independent of weight [28]. Epidemiological evidence shows how the Mediterranean diet impacts MASLD; a healthy plant-based diet, nut consumption and adherence to the Mediterranean dietary pattern are all associated with either a lower MASLD prevalence or a lower risk [52,53,54,55]. Similarly, a meta-analysis including over 1.2 million participants showed that a high vegetable intake reduced the risk of hepatocellular carcinoma by 28%, a notable complication of advanced MASLD [56]. Overall, there is potential benefit with long-term adherence to the Mediterranean-LCD in people with obesity and MASLD. However, further evaluation is required via larger randomised controlled trials to assess its impact on IHL and liver fibrosis, determined via more robust measures.

### 4.3. Green Mediterranean-LCD

Our review shows the potential efficacy of the green-Mediterranean-LCD on liver health. The green-Mediterranean diet is rich in dietary polyphenols, unsaturated fats and plant-based food whilst being low in red and processed meats [27,57]. In the DIRECT-PLUS trial, a high polyphenol intake was achieved via three to four cups of green tea per day, walnuts and a mankai green shake [22]. The trial reported that the gut microbiota may underpin the Green-Mediterranean diet’s impact on liver health. In the DIRECT-PLUS study, those adhering to the green-Mediterranean-LCD had distinct changes in the gut microbiome [58]. Rodent models show that gut dysbiosis contributes towards gut vascular barrier damage, leading to bacterial translocation into the circulation and, consequentially, liver inflammation [59]. Through its influence on metabolism, the microbiota composition may influence pro-inflammatory and anti-inflammatory pathways, thereby benefiting or worsening liver health [60]. Mechanistically, even one day of dietary change alters the gut microbiota, highlighting how the long-term dietary pattern influences health [61]. Overall, given the links delineated between the gut microbiota, lifestyle intervention and MASLD, further evidence is required to allow for the advancement of this novel therapeutic strategy [18].

### 4.4. Weight Loss for MASLD

Our review shows the clear benefit of calorie restrictions for liver health and body anthropometry in patients with MASLD and obesity. Weight reduction interventions for MASLD are an emerging paradigm. The BRAVES trial randomised patients with MASH and obesity to either lifestyle intervention, Roux-en-Y-gastric bypass (RYGB) or laparoscopic sleeve gastrectomy (LSG). The metabolic surgery groups reduced body weight significantly and had a 3.5 times greater probability of MASH resolution and improvements in liver enzymes compared to lifestyle intervention [62]. Similarly, there is potential for GLP-1 receptor agonists (liraglutide, semaglutide, tirzepatide, retatrutide) to benefit MASLD. In a 48-week phase two trial of 2.4 mg semaglutide vs. placebo in patients with MASH cirrhosis, the average weight loss with therapy was −8.75 kg (−5.09, −12.41 kg) vs. the placebo. Semaglutide did not significantly benefit liver fibrosis (via biopsy); however, it significantly reduced liver steatosis (via MRI-PDFF), ALT and AST [63]. Perhaps with more effective therapies (tirzepatide, retatrutide), liver fibrosis will be impacted. Overall, the clear relationship between body weight reduction and liver health provided the rationale for this review. However, based on our analysis, it is clear that it is not only energy restriction that is important, but also the type of foods consumed. 

### 4.5. Future Research

Our review points towards future MASLD dietary intervention priorities for clinical trials. First, our review finds that no studies were conducted in Black African populations, and only one study, STANDBY, was conducted in a South Asian population [40]. Different cultures may find different dietary interventions acceptable; for instance, in a recent qualitative study, TDR was considered unacceptable for people with T2D of South Asian ethnicity [64]. This is important, as an unacceptable intervention will negatively impact patient experience, retention and, therefore, clinical outcomes. Second, our systematic review highlights that more male patients were recruited to trials. Further research must ensure that females with MASLD and obesity are recruited. Third, recruitment to TDR trials of MASLD and obesity should focus on those with prediabetes or normoglycaemia. This is because the two trials of TDR were of patients with T2D. Finally, our review highlights that only one small study has analysed liver histology outcome data in people with MASLD and obesity. Given that liver histology is the gold standard outcome measure for MASLD, research should ensure that this outcome measure is included [65]. These measures will improve the external validity of further data, ensuring translatability to diverse populations. Finally, all trials should focus on long-term outcome data. 

### 4.6. Strengths & Limitations

Our systematic review has several strengths. First, we analysed the effect of various types of LCD in people with MASLD and obesity. This allowed us to assess how calorie restrictions and food compositions impact outcomes. Second, we used a rigorous search strategy of three different databases whilst searching the reference lists of relevant studies. Third, all but two studies included were randomised controlled trials, which minimises the risk of bias. Finally, two reviewers independently reviewed abstracts and full texts and performed quality assessment and data extraction, reducing the risk of bias in our findings.

Our review does have limitations. First, given that lifestyle interventions can be relatively heterogeneous in their approach, i.e., including a physical activity prescription, this made data integration challenging. Second, the outcome data in certain studies were not reported in a way that could be meta-analysed. Third, our review did not highlight any studies of people of Black African or Caribbean ethnicity, and only one highlighted individuals of South Asian ethnicity, meaning our results have limited external validity when applied to these populations. Fourth, most trials included middle-aged people, meaning more trials are required in people who are of younger and older ages. Fifth, there were no TDR data in people with MASLD and obesity but without T2D. Sixth, only one small study included liver histology data. Liver histology is the gold standard assessment for MASLD, so further trials are required to assess this to confirm efficacy. 

## 5. Conclusions

Overall, our systematic review shows that in those with MASLD and obesity, the LCD reduces body weight and liver fat content with a trend towards improved serum transaminase levels. Furthermore, in those with MASLD, obesity and recently diagnosed T2D, TDR reduces IHL. We also delineate that the consumption of a Mediterranean LCD for 12 months or more, or of a green-Mediterranean LCD, may reduce IHL and ALT, independent of weight, versus other LCDs. Future clinical trials should evaluate whether TDR, potentially with added dietary polyphenols, impacts liver health in those with MASLD and obesity. They must also ensure the diverse enrolment of participants, particularly from Black African and Caribbean and South Asian populations, as these demographics are currently underrepresented. 

## Figures and Tables

**Table 1:** Dietary Interventions.

Intervention	Definition/Description
Low-Calorie Diet (LCD)	One of (A) 800–1500 kcal/day; (B) 500 kcal/day calorie reduction or (C) 25% reduction in calories from baseline
Very-Low-Calorie Diet (VLCD)	One of: (A) 450–800 kcal/day; (B) 1200 kcal/day calorie reduction; (C) 60% reduction in calories from baseline
Total-Dietary Replacement (TDR)	Very-Low-Calorie Diet utilising nutritionally complete meal replacements. This includes 3 months of total-meal replacement followed by structured food reintroduction for 2 to 8 weeks.
Mediterranean Low-Calorie Diet (MED-LCD)	Low-Calorie Diet, which follows the Mediterranean dietary pattern, i.e., a minimal consumption of processed/ultra-processed foods and a high consumption of whole grains, nuts, legumes, olive oil, vegetables and fruits.
Green Mediterranean Low-Calorie diet (GREEN-MED-LCD)	Low-Calorie Diet, which follows a green Mediterranean dietary pattern, i.e., a minimal consumption of processed/ultra-processed foods and a high consumption of whole grains, nuts, legumes, olive oil, vegetables and fruits. In addition, this is supplemented with foods high in dietary polyphenols, i.e., walnuts, green tea and Mankai.
Low-Carbohydrate Diet	Low-Calorie Diet which limits carbohydrate intake to <20 g/day.
Low-Calorie Diet—Polyunsaturated fatty acids	Low-Calorie Diet enriched in polyunsaturated fatty acids.
Low-Calorie Diet—Monounsaturated fatty acids	Low-Calorie diet enriched in monounsaturated fatty acids.
Very-Low-Calorie Ketogenic Diet	VLCD for 2 months, inducing ketogenesis. Consists of LCD (600–800 kcal/day) and is low in carbohydrates (<50 g/day) and lipids (10 g of olive oil/day)

**Table 2:** Inclusion and Exclusion Criteria.

Inclusion Criteria	Exclusion Criteria
Randomised Controlled trial or cohort study	Not original research (excluding reviews, case reports and practice guidelines)
Participants Aged ≥ 18 years old	Not a Human Study
Presence of NAFLD or MASLD *	Does not report primary and/or secondary outcomes
Participants with overweight or obesity	No Comparator Group (i.e., control or alternative dietary intervention)
Reported in English	Non-English language publication
Treated with a Low-Calorie Diet or Very-Low-Calorie Diet Intervention	

* MASLD is a form of steatotic liver disease, which includes having hepatic steatosis on imaging or biopsy with at least one of the five cardiometabolic diagnostic criteria [2]: MASLD: Metabolic-dysfunction associated Steatotic Liver Disease, NAFLD: Non-Alcoholic Fatty Liver Disease.

**Table 3:** Summary of the Included Studies.

Comparison		Study	Design	N	Duration (Months)	Location	Sex (% Male)	Age (Years ± SD)	Ethnicity
LCD vs. Control	Non-TDR	Asghari, 2022 [41]	RCT	60	3 m	Iran	65% M	39.7 ± 6.3 y	Middle-Eastern ^
		Chen, 2020 [36]	RCT	44	2 m	China	63.6% M	38.1 ± 9.4 y	East Asian ^
		Elias, 2010 [37]	Cohort	31	6 m	Brazil	48.3% M	47.5 ± 11.6 y	Hispanic and Latino ^
		El-Kader, 2016 [38]	RCT	100	3 m	Saudi Arabia	70% M	51.0 ± 5.6 y	Middle Eastern ^
		Promrat, 2009 [39]	RCT	31	11 m	USA	70.9% M	48.5 ± 11.3 y	84% Caucasian
	TDR	Taylor, 2018 DIRECT [12,13]	RCT	90/306 ^a^	12 m	UK	57.8% M	52.8 ± 7.9 y	98.3% Caucasian
		Sattar, 2022 STAND-BY [40]	RCT	25	3 m	UK	52% M	45.8 ± 11.1 y	South Asian
MED-LCD vs. Control or other LCD		Gepner, 2018/2019 CENTRAL [28,29]	RCT	278	18 m	Israel	89% M	47.9 ± 9.3 y	Middle Eastern ^
		Marin-Alejandre, 2019/2021FLiO [30,31]	RCT	98	6–24 m	Spain	52% M	50.1 ± 9.3 y	Caucasian ^
		Montemayor, 2022 FLIPAN [26]	RCT	128 ^b^	12 m	Spain	63.3% M	52.9 ± 7.27 y	Caucasian ^
		Ristic-Medic, 2020 [34]	RCT	27	3 m	Serbia	100% M	33.6 ± 4.2 y	Caucasian ^
Green-MED-LCD vs. MED-LCD or HDG		Meir, 2019/2021 DIRECT-PLUS [22,27]	RCT	294	18 m	Israel	88.1% M	51.1 ± 10.5 y	Middle Eastern ^
Low-Carb LCD vs. LCD		Browning, 2011 [32]	Non-RCT *	18	0.5 m	USA	27.8% M	44.5 ± 11.5 y	Not Stated
VLCKD vs. LCD		Cunha, 2020 [33]	RCT	46	2 m	Brazil	17.4% M	40.3 ± 11.3 y	Hispanic and Latino ^
LCD MUF vs. PUF		Aller, 2014 [35]	RCT	44	3 m	Spain	28.1% M	49.3 ± 16.7 y	Caucasian ^

Table describing the characteristics of the included studies. LCD, Low-Calorie Diet; RCT, Randomised Controlled trial; m, months; M, Male; y, years; SD, standard deviation; N, number; MED-LCD, Mediterranean Low-Calorie Diet; TDR, Total-Dietary Replacement; Green-MED-LCD, Green-Mediterranean Low-Calorie Diet; VLCKD, Very-Low-Calorie Ketogenic Diet; MUF, Monounsaturated Fatty Acids; PUF, Polyunsaturated Fatty Acids. * Browning et al. [32] assigned participants to a Low-carbohydrate diet or LCD in a semi-random manner; therefore, it is not an ‘RCT’. ^ Ethnicity is not explicitly stated in the paper; however, the participants are likely predominantly of this ethnicity based on the geographical location and the details from the methods. ^a^: For the DIRECT trial, 306 participants took part in the full study, and 90 of the participants took part in the detailed metabolic studies. ^b^ For the FLIPAN study, 155 participants were randomised, and data were analysed for 128 participants.

## Data Availability

The data analysed in this systematic review are available in the original publications and in this manuscript’s Supplementary Materials.

## Supplementary Materials

The following supporting information can be downloaded at: https://www.mdpi.com/article/10.3390/nu16071030/s1, Supplementary Methods S1: Search Strategy for Database; Supplementary Figure S1: Quality Assessment; Supplementary Figure S2: LCD ALT Funnel Plot; Supplementary Figure S3: LCD AST Funnel Plot; Supplementary Figure S4: LCD Body Weight Funnel Plot; Supplementary Figure S5: LCD Intrahepatic Liver Fat Funnel Plot; Supplementary Figure S6: Mediterranean-LCD Body Weight Funnel Plot; Supplementary Figure S7: Mediterranean-LCD ALT Funnel Plot; Supplementary Figure S8: Mediterranean-LCD AST Funnel Plot; Supplementary Table S1: Included Study Characteristics; Supplementary Table S2: Demographic Characteristics of the Included Studies; Supplementary Table S3: Studies Nearly Meeting Inclusion; Supplementary Results S1.
