# Supplementary material for: Effect of a Low-Calorie Dietary Intervention on Liver Health and Body Weight in Adults with Metabolic-Dysfunction Associated Steatotic Liver Disease (MASLD) and Overweight/Obesity: A Systematic Review and Meta-Analysis
Source: Nutrients. 2024 Apr 1;16(7):1030. doi: 10.3390/nu16071030 (PMC11013586; doi:10.3390/nu16071030)
Supplement: Supplementary file 1 [file nutrients-16-01030-s001.zip › nutrients-2902027-supplementary.pdf]

Supplementary Materials

Effect of a Low-Calorie Dietary Intervention on Liver Health and Body Weight in Adults with Metabolic-Dysfunction Associated Steatotic Liver Disease (MASLD) and Overweight/Obesity: A Systematic Review and Meta-Analysis

Laurence J. Dobbie [1,2,†], Jamie Burgess [1,3,†], Azlinda Hamid [1], Sarah J. Nevitt [4,5], Theresa J. Hydes [1,3], Uazman Alam [1,3] and Daniel J. Cuthbertson [1,3,*]

Supplementary Methods S1: Search Strategy for Databases

1. "NON-ALCOHOLIC FATTY LIVER DISEASE"/
2. ("non alcoholic fatty liver disease" OR "non-alcoholic fatty liver disease" OR "NAFLD" OR "metabolic associated liver disease" OR "non-alcoholic steatosis" OR "non-alcoholic steatohepatitis") ti.ab
3. (1 OR 2)
4. "CALORIC RESTRICTION"/
5. "("low-calorie diet*" OR "low calorie diet*" OR "LCD" OR "800kcal-1500kcal" OR "800kcal - 1500kcal" OR "800 to 1500 calories" OR"800 calories to 1500 calories" OR "meal replacement*" OR "isocaloric diet*") ti,ab
6. (diet OR diets).ti,ab
7. ("low fat" OR "low-saturated fat" OR "low saturated fat" OR "low carbohydrate" OR "low carb*") ti.ab
8. (6 AND 7)
9. (4 OR 5 OR 8)
10. (3 AND 9)
11. "GASTRIC BYPASS"/
12. ("Gastric Bypass" OR "weight loss surgery" OR "gastric band" OR "gastric sleeve" OR "Roux-en-Y bypass") ti.ab
13. (11 OR 12)
14. (3 AND 13)

Figure S1: Quality Assessment

(A) Cochrane Risk of Bias Tool Assessment of randomised controlled trials

Study ID	Experimental	Comparator	D1	D2	D3	D4	D5	Overall
Aller, 2014	LCD - high monounsaturated	LCD - high polyunsatured diet	!	!	+	+	!	!
Asghari, 2022	LCD	Control	+	+	+	+	+	+
Chen, 2020	Low Carb High Fibre	Control	+	-	+	-	!	-
Cunha, 2020	VLCKD	LCD	+	!	+	+	!	!
El-Kader, 2017	Lifestyle modification	Control	!	-	!	+	!	-
Gepner, 2019	Green-MED / MED	LFD	+	+	+	+	+	+
Marin-ALejandre, 2019	FLiO	AHA	!	+	+	+	!	!
Meir, 2021	MED	HDG	+	+	+	+	+	+
Montemayor, 2022	MED	LCD	+	+	+	+	+	+
Promrat, 2009	Lifestyle intervention	Control	+	!	+	+	!	!
Ristic-Medic, 2020	MED	LFD	+	+	+	+	!	+
Sattar, 2022	LCD	Control	+	+	+	+	+	+
Taylor, 2018	LCD	Control	+	+	+	+	+	+

+ Low risk
! Some concerns
- High risk

D1 Randomisation process
D2 Deviations from the intended interventions
D3 Missing outcome data
D4 Measurement of the outcome
D5 Selection of the reported result

(B) ROBINS-I Tool assessment of non-randomised studies

Study	Bias due to confounding	Bias in selection of participants into the study	Bias in classification of interventions	Bias due to deviations from intended interventions	Bias due to missing data	Bias in measurement of outcomes	Bias in selection of the reported result	Overall Bias
Browning, 2011	Serious	Low	Low	Serious	Low	Serious	Moderate	Serious
Elias, 2010	Serious	Moderate	Serious	Critical	Low	Serious	Low	Critical

Reference: [12,13,22,26–40]

Figure S2: LCD, ALT Funnel Plot

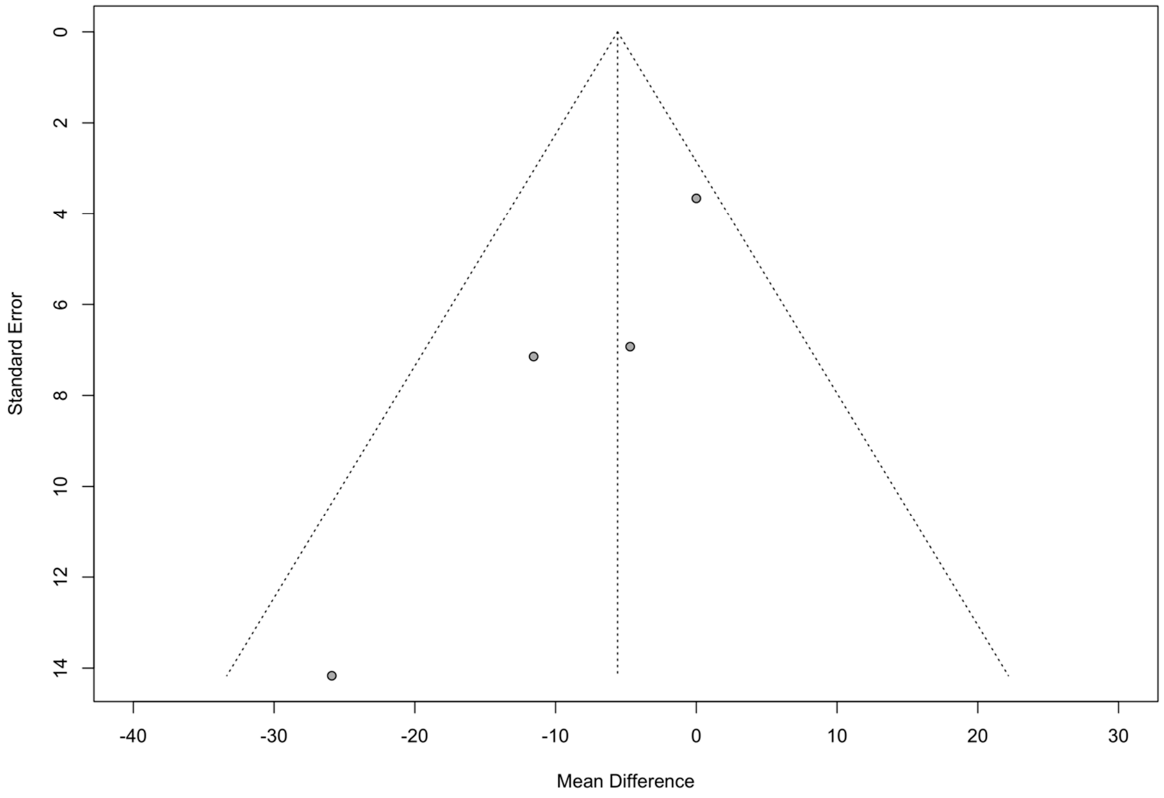

Figure S3: LCD AST Funnel Plot

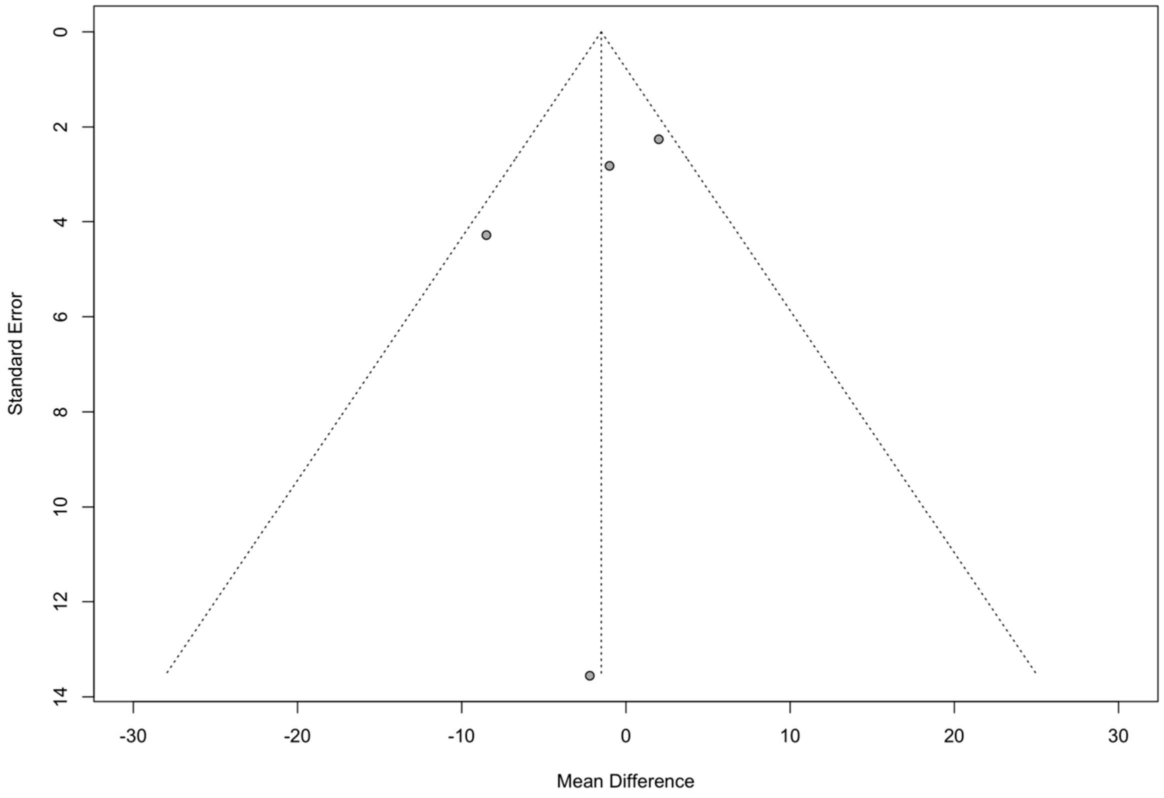

Figure S4: LCD Body Weight LCD Funnel Plot

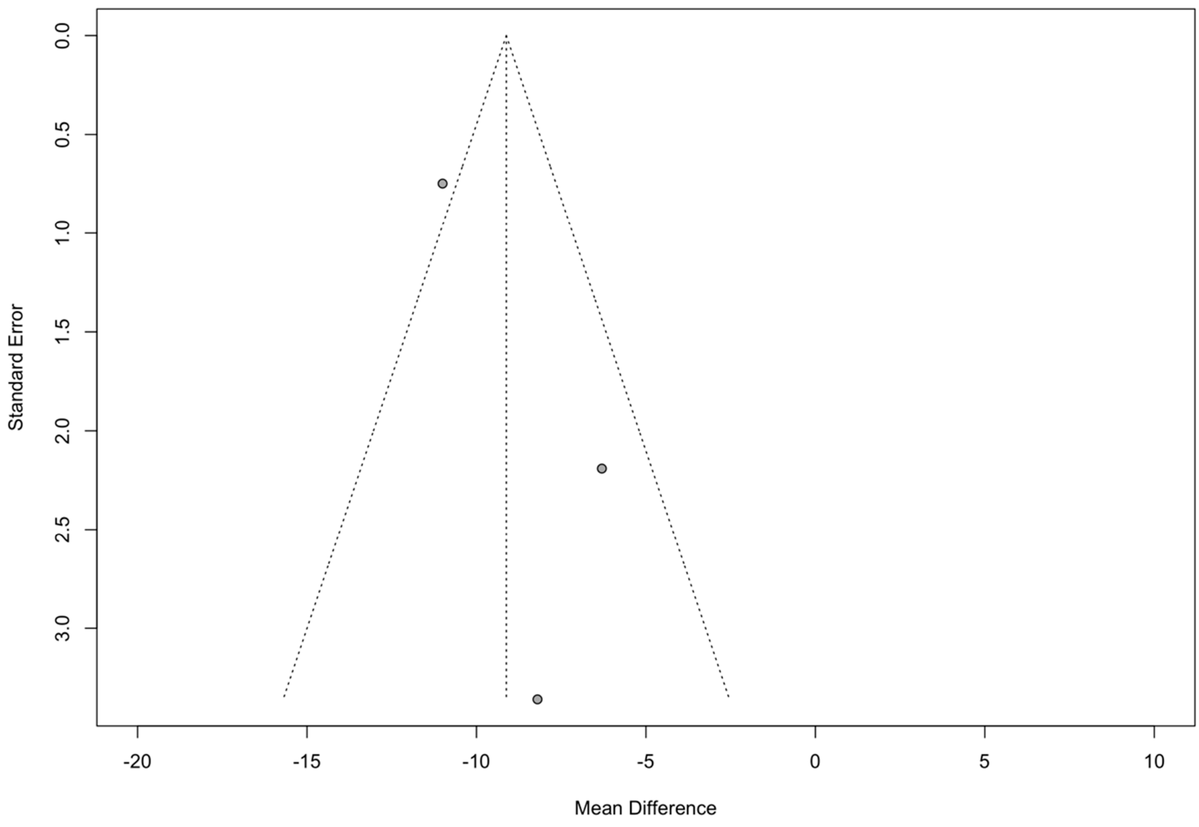

Figure S5: LCD Intrahepatic Liver Fat Funnel Plot

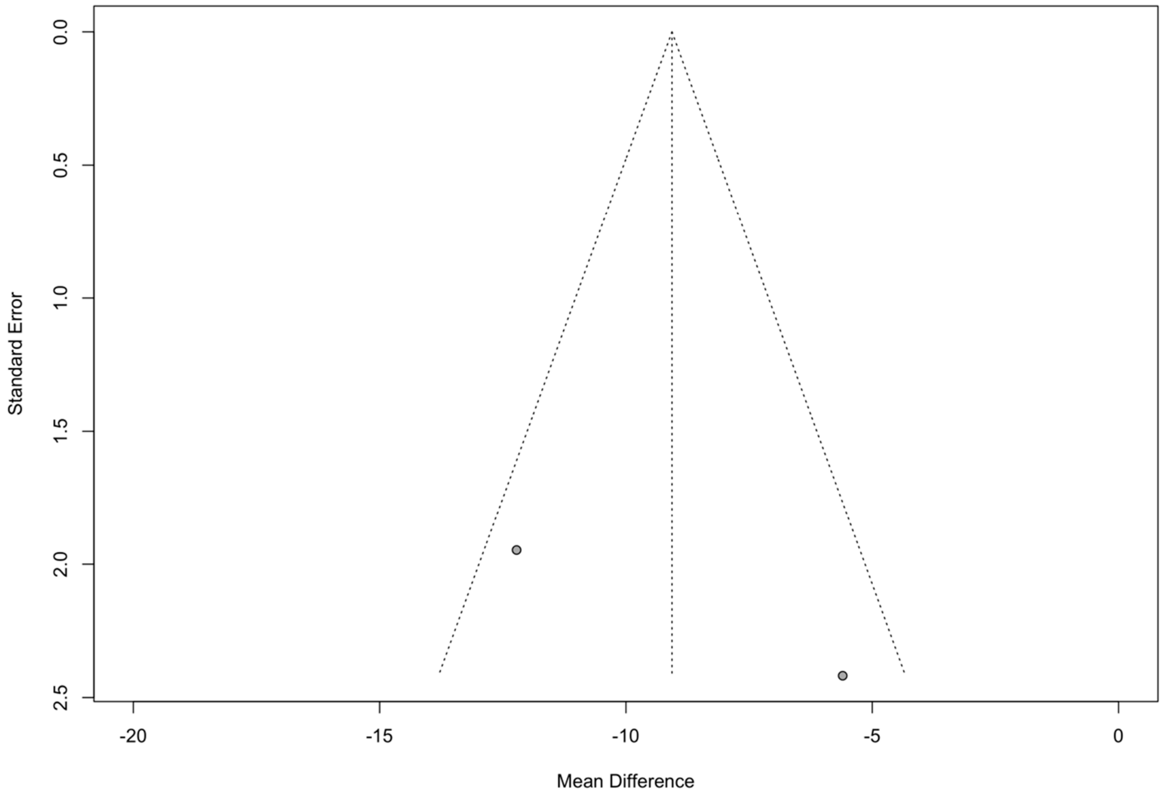

Figure S6: Mediterranean-LCD Body Weight Funnel Plot

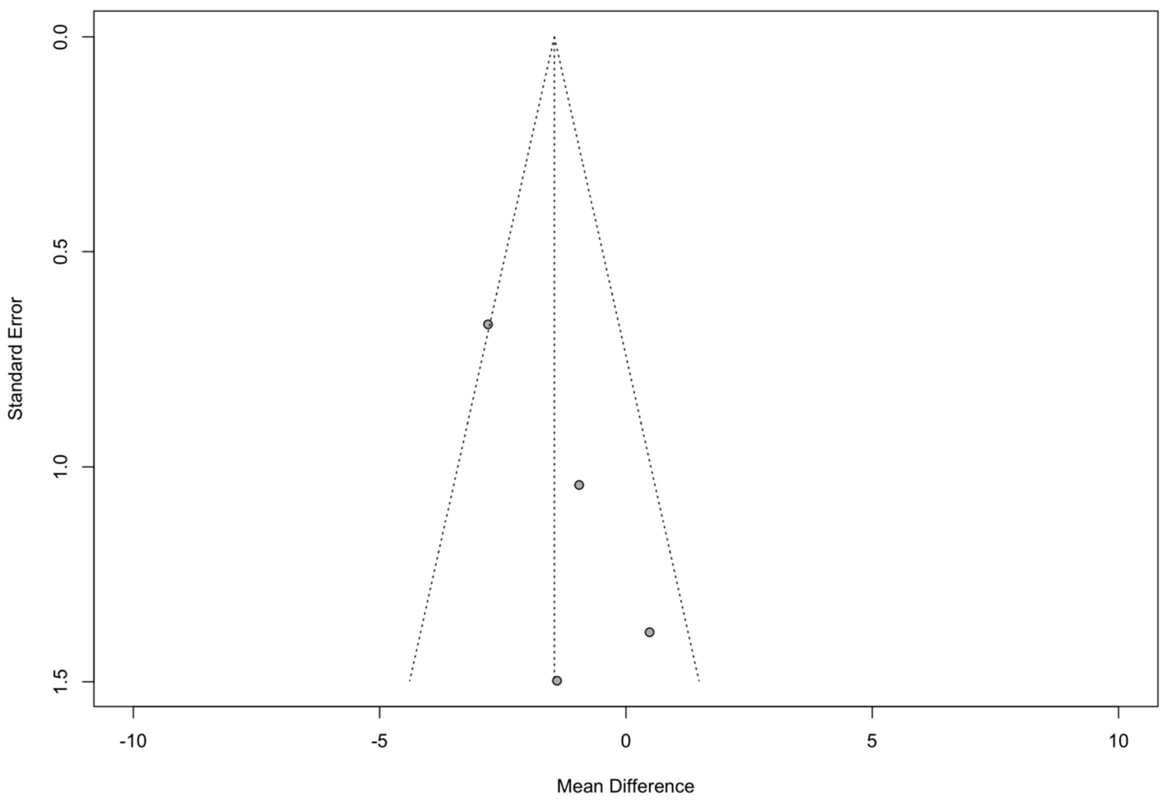

Figure S7: Mediterranean-LCD ALT Funnel Plot

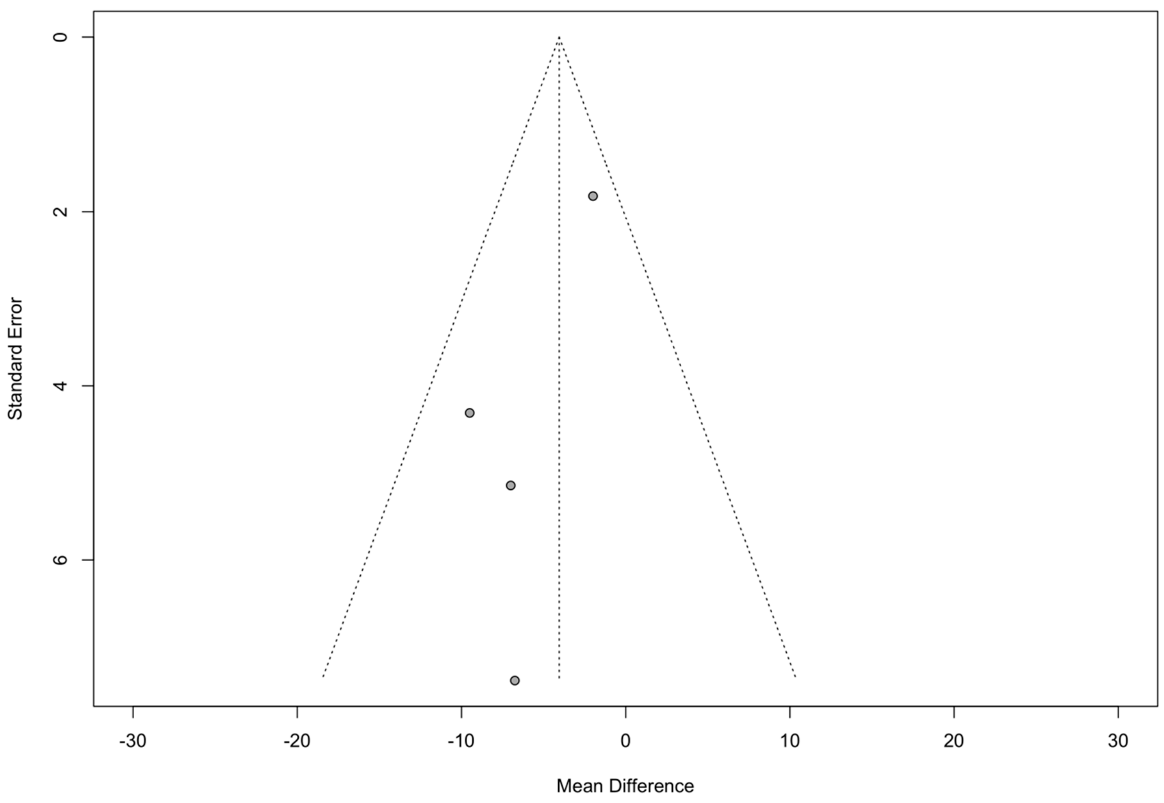

Figure S8: Mediterranean-LCD AST Funnel Plot

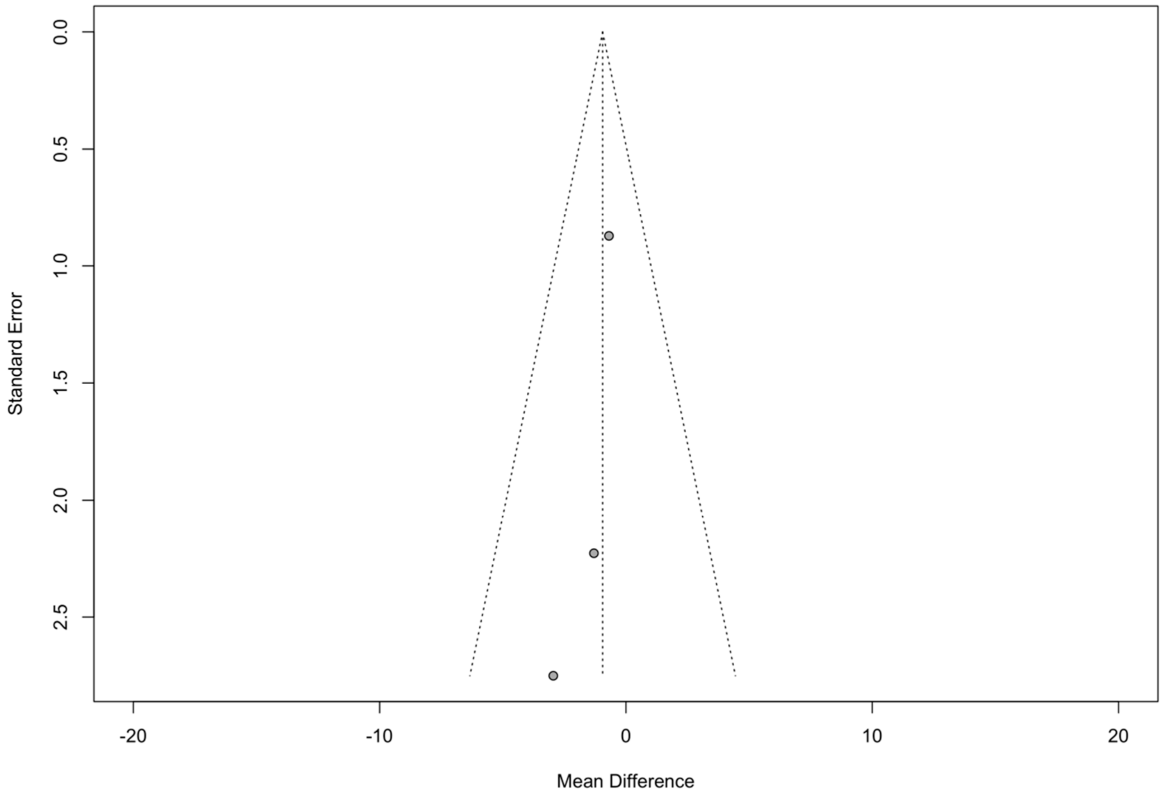

Table S1: Included Study Characteristics

Study	Design	Study Arms	Recruitment Source	Study Arm 1	Study Arm 2	Study Arm 3 (or Arm 2 cont)
**LCD vs Control**						
Asghari, 2022 [41]	Iran<br>RCT<br>01/2016 – 06/2016	LCD vs control<br>Arm-1: Control<br>Arm-2: LCD	Golgasht Outpatient Clinic University of Tabriz (Iran)	**Control Group**<br><br>Healthy eating and weight control advice.	**LCD Group**<br><br>Calorie restriction treatment.<br>Targeted to lose a maximum of 10% of their baseline body weight.<br>To restrict calories, 500 to 1000 kcal/day (depending on body weight) was deducted from the estimated energy requirements.<br>A dietitian fully informed participants of both groups about the healthy eating fundamentals.<br>Individual interviews during follow-up visits every two weeks to assess adherence to the prescribed diet plan, discuss and resolve any barriers or concerns related to the diet and encourage compliance.<br><br>Energy Composition<br>17% protein<br>30% fat<br>53% carbohydrate	
2020, Chen [36]	China<br>RCT<br>10/2015 – 05/2017	LCD vs Control<br>Arm 1: Control<br>Study Arm 2: LCD	A clinic of Huadong Hospital affiliated to Fudan University	**Control**<br><br>Face-to-face counselling with lifestyle guidance and weekly phone call from dietician	**LCD**<br><br>1000-1200kcal/day<br>400g/day vegetables<br>1800ml/day water<br><br>Energy Composition<br>20-25% carbohydrates<br>40-45% Protein (beans, nuts, soy milk, chicken and fish)<br>30-35% Fats, n-3 and n-6 polyunsaturated fatty acids<br>Low saturated fatty acid intake.<br>5g/day dietary fibre supplement<br>Calcium + magnesium tablets 2x/day	

					Multivitamins 2x/day<br>Low Glycaemic Index
Elias, 2010<br>[37]	Brazil<br>Cohort<br>2005 - 2008	Adherent vs non-adherent to LCD<br><br>Arm 1: Non-adherent<br>Arm 2: Adherent	Gastroenterology outpatient clinic of the Federal University of Sao Paulo	**Non-adherent to LCD**<br><br><5% weight loss defined those as non-adherent to LCD.	**Adherent to LCD**<br><br>500-1000kcal/day Reduction<br>Nutritional instructions according the clinical guidelines from National Institute of Health and Northern American Association for Study of Obesity<br><br>Energy Composition<br>55% Carbohydrates<br>30% Total Fat<br>8-10% Saturated fats<br>>15% Monounsaturated fats<br>>10% Polyunsaturated fats<br>15% Protein<br><300mg/day Dietary Cholesterol<br>20-30g/day Fibre
El-Kader, 2016<br>[38]	Saudi Arabia<br>RCT	Lifestyle vs control<br><br>Arm 1: Control<br>Arm 2: Lifestyle	Internal Medicine Department at King Abdul Aziz University Hospital and other Hospitals at Jeddah Area	**Control**<br><br>Maintain ordinary current lifestyle	**Lifestyle Intervention**<br><br>Energy Intake: 1200kcal/day<br>Exercise training programme: treadmill based training programme at 65-75% of maximal HR, 30min sessions, 3x/week<br><br>Energy Composition<br>50-55% Carbohydrates<br>30-35% Fat<br>15% Protein

Promrat, 2009 [39]	USA<br>RCT<br>01/2005 – 02/2007	Lifestyle vs Control<br><br>Arm 1: Control<br>Arm 2: Lifestyle	Newspaper advertisement and local physicians in Rhode Island, USA	**Control**<br><br>Small group sessions about NASH and principles of healthy eating, physical activity and weight control, every 12 weeks.	**Lifestyle Intervention**<br><br>Assigned a calorie goal based on starting weight: 1000-1200 kcal/day if baseline weight <200 lb or 1200-1500/day if baseline weight >200 lb.<br>Dietary recommendations in this study were consistent with the recommendations of the American Heart Association, the American Diabetic Association, and the American College of Sports Medicine.<br><br>Weight loss lifestyle intervention based on Diabetes Prevention Program and Look AHEAD. 7-10% weight loss goal in 6 months, then WL maintenance.<br>Small group nutritional sessions every week for 6 months, then biweekly for months 7-12.<br>Also adhered to Portion Controlled Diet and Higher Exercise goals<br><br>Energy Composition<br>25% Fat
**TMR vs Control**					

DIRECT (metabolic substudy) Taylor, 2018 [12,13]	UK<br>Cluster RCT<br>07/2014 – 08/2017	LCD vs Control<br><br>Arm 1: Control<br>Arm 2: LCD	Recruited via their general practices	**Control**<br><br>Continued usual diabetes management by their GP practice according to current UK clinical guidelines.	**LCD**<br><br>Stopped all anti-diabetic medication on day 1 of the Counterweight Plus weight management programme consisting of 825–853 kcal/d liquid formula diet (Cambridge Weight Plan Ltd., UK) continued for 12-20 weeks, followed by a 2-6 week food reintroduction phase, then ongoing support for weight maintenance.<br><br>Total diet replacement phase using a low energy formula diet for 3-5 months<br>825–853kcal/day<br>59% carbohydrate<br>13% fat<br>26% protein<br>2% fibre<br><br>Structured food reintroduction of 2–8 weeks<br>50% carbohydrate<br>35% total fat<br>15% protein<br><br>Step counters were provided at the start of food reintroduction, and physical activity strategies were introduced, to help participants in the intervention group to reach and maintain their individual sustainable maximum—up to 15 000 steps per day.
STANDby, Sattar, 2022 [40]	UK<br>RCT with observational follow-up<br>02/2019 – 07/2021	Immediate LCD vs Delayed LCD<br><br>Arm 1: Control<br>Arm 2: LCD<br><br>Further analysis of the delayed LCD group, who went on to have LCD	Recruited via general practice	**Delayed TDR**<br><br>Usual care, managed under current NHS clinical guidelines, for three months before returning to receive the intervention. Asked to maintain weight in the initial control period, and wait until TMR commenced.	**Immediate TDR**<br><br>Counterweight Plus programme which was used in the DiRECT trial.<br>Delivered by dietitians experienced in Counterweight Plus programme delivery, comprised a Total Diet Replacement phase (TDR: an 825–853 kcal/day formula diet) for 3–5 months (flexible duration to accommodate participants with commitments or practical limitations due to COVID-19), and then stepped food reintroduction (FR) for six-eight weeks.<br><br>On completion of FR period participants were given advice, and written information, on maintaining weight loss, and returned to the care of their GP, who was informed of the trial and their patient's progress.

MED-LCD vs LCD					
CENTRAL<br>Gepner, 2019<br>[28,29]	Israel<br>RCT<br>10/2012 – 04/2014	MED LCD vs AHA LCD<br>Arm 1: Control AHA LCD<br>Arm 2: MED LCD	Isolated research centre workplace in Israel	**AHA Low-Fat LCD**<br>(High carbohydrate + Low-saturated fat diet)<br><br>Calorie restricted<br>6 months in 50% randomised to PA, 50% no-PA.<br>Nutritional counselling: whole grains, vegetables, fruits and legumes, limits consumption of additional fats, sweets, high-fat snacks.<br><br>**Energy Composition**<br>Total Fat: <= 35%<br>Saturated Fat: <= 7-10% Dietary cholesterol: <= 300mg trans fats not recommended<br>Carbohydrates: > 55%, Not restricted.<br>Fibre: increased whole grains.	**MED-LCD**<br>(Low-carbohydrate + high mono-unsaturated fat diet)<br><br>Calorie restricted<br>6 months in 50% randomised to PA, 50% no-PA.<br>Nutritional counselling: Rich in vegetables and legumes, and low in red meat, with poultry and fish replacing beef/lamb.<br><br>Energy Composition<br>Carbohydrate: <40gr/day for 2 months, then gradually increasing to <80gr/day<br>Total Fat: liberal, expected >40%<br>Saturated Fat: Not specifically restricted<br>Dietary Cholesterol: not specifically restricted.<br>Trans Fat: not recommended<br>45gr/day Virgin Oliver Oil (at least 20% monounsaturated fat)<br>25gr/day nuts<br>Fish 2x/week<br>28g/day walnuts (160kcal), starting 3rd month
FLiO<br>Marin-Alejandre, 2019/2022 (Nutrients / Liver International)<br>[30,31]	Spain<br>RCT<br>06/2016 – 06/2018	FLiO vs AHA<br>Arm 1: Control AHA LCD<br>Arm 2: FLiO LCD (MED-LCD)	Individuals with confirmed hepatic steatosis via USS in Navarra, Spain.	**AHA LCD**<br><br>30% energy restricted LCD<br>10,000 steps/day activity recommendation<br>7 day menu plan<br><br>Energy Composition<br>3-5 meals/day<br>Carbohydrates: 50-55% total kcal<br>Protein: 15% total kcal<br>Lipids: 30% total kcal	**FLiO (MED-LCD with high-meal frequency)**<br><br>30% energy restricted diet<br>10,000 steps/day activity recommendation<br>7 day menu plan<br><br>Energy Composition<br>7 meals/day -> breakfast, lunch, dinner, 2 snacks AM + PM<br>Carbohydrates: 40-45% kcal (low GI)<br>Proteins: 25% kcal (mainly vegetable source)<br>Lipids: 30-35% kcal (extra virgin olive oil + Omega-3 fatty acids rather than saturated / trans-fats)

				Healthy Fatty Acid Profile	High adherence to Mediterranean diet	
FLIPAN<br>Montemayor, 2022<br>[26]	RCT<br>Spain<br>06/2018 – 01/2020	LCD vs MED-HMF vs MED-PA<br><br>Arm 1: Conventional Diet (CD)<br>Arm 2: MED-high meal frequency<br>Arm 3: MED-with physicial activity	Not Stated	**Control**<br><br>American Association for the Study of Liver Disease (AASLD) recommendations:<br>Energy restriction enough to lose 3–5% of body weight to improve steatosis, and 7–10% to improve most of the histopathological features of NAFLD/NASH, including fibrosis<br><br>Energy Composition<br>20–35% fat<br>10–35% protein<br>45–65% carbohydrate<br>Fiber 25 g/day<br>Cholesterol <250 mg/day<br><br>Minimum 10,000 steps/day (recorded via pedometer)	**Mediterranean Diet–high meal frequency (MD–HMF)**<br><br>Energy Composition<br>30–35% fat (mainly mono- and poly-unsaturated fatty acids from extra virgin olive oil, nuts, and omega-3 containing foods)<br>25% protein (mainly from vegetable sources)<br>40–45% carbohydrates (50–70% of the total carbohydrate intake should be low glycemic index and rich in fiber).<br>-7 meals/day, gradual reduction of caloric content at each main meal, with the highest calorie meals to be consumed early during the day (breakfast, lunch, dinner and two snacks in the morning and two snacks in the afternoon).<br><br>Minimum 10,000 steps/day (recorded via pedometer)	**Mediterranean Diet–physical activity (MD–PA)**<br><br>Energy-restricted MedDiet.<br>meal frequency was of 4–5 meals a day including snacks.<br><br>Energy Composition<br>-35–40% fat (8–10% of saturated fatty acids, >20% of monounsaturated fatty acids, >10% of polyunsaturated fatty acids and <300 mg/day of cholesterol)<br>-20% proteins<br>40–45% carbohydrates (low GI)<br>-Sodium chloride <6 g/day<br>-Dietary fiber >30–35 g/day<br><br>35 min interval training session three times a week, with a combination of two instructor-led on-site training sessions and one remote prescribed training session a week for the whole duration of the trial. Physical activity sessions of 35 min consisted of 5 min warm-up, 20 min interval training, and 10 min breathing and stretching.
Ristic-Medic, 2020<br>[34]	Serbia<br>RCT<br>02/2013 – 03/2015	MED LCD vs LFD<br><br>Arm 1: LFD<br>Arm 2: MED LCD	Centre of Research in Nutrition and Metabolism, Institute for Medical Research	**LFD**<br><br>600-800kcal/day reduction (30% reduction)<br>Personalised Nutritional Counselling<br>30min/day walking increase	**MED-LCD**<br><br>600-800kcal/day kcal reduction<br>Personalised Nutritional Counselling<br>30min/day walking increase<br>Target for 5% initial body weight loss.<br>Breakfast, lunch, dinner, one morning snack, one afternoon snack.	

				Target for 5% initial body weight loss.<br>Breakfast, lunch, dinner, one morning snack, one afternoon snack.<br><br>Energy Composition<br>Carbohydrates: 60% kcal (low Glycaemic index)<br>Protein: 15% kcal<br>Lipids: 25% kcal<br>Balanced in micronutrient content (vitamins + minerals)	Energy Composition<br>Carbohydrates: 50% kcal<br>Proteins: 15% kcal<br>Lipids: 30% kcal<br>Balanced in micronutrient content (vitamins + minerals)	
**GREEN-MED LCD**						
DIRECT-Plus<br>Tsaban, 2021<br>Meir, 2021<br>[22, 27]	Israel<br>RCT<br>05/2017 – 11/2018	MED vs Green-MED vs Control<br>Arm 1: HDGl<br>Arm 2: MED LCD<br>Arm 3: Green-MED LCD	Isolated workplace at Nuclear Research Centre Negev, Dimona, Israel	**Healthy Dietary Group (HDG)**<br><br>Standard nutritional counselling<br>18 month gym membership: 45-60mins aerobic exercise + resistance training 3-4/week<br>Lifestyle Group Sessions<br><br>Dietary Advice: Limit dietary cholesterol, trans-fat, saturated-fat, sugars, and salt. Increase intake of vegetables.	**Mediterranean Diet**<br><br>Calorie restricted Mediterranean diet<br>Women: 1200-1400kcal/day<br>Men: 1500-1800kcal/day<br>18 month gym membership -> 45-60mins aerobic exercise + resistance training 3-4/week<br>Lifestyle Group Sessions<br><br>Energy Composition<br>Fat: ~40% (mainly PUFA and MUFA)<br>Carbohydrates: <40g/day in 1st 2 months, then increased up to 80g/day.<br>Less/avoid red + processed meats.<br>Polyphenols: 440mg/day (from 28g/day walnuts)<br>Reduced Poultry intake<br><br>Dietary guidance: Limit dietary cholesterol, trans-fat, saturated-fat,	**Green Mediterranean Diet**<br><br>Calorie restricted Green Mediterranean diet – richer in plants and polyphenols<br>Women: 1200-1400kcal/day<br>Men: 1500-1800kcal/day<br>18 month gym membership -> 45-60mins aerobic exercise + resistance training 3-4/week<br>Lifestyle Group Sessions<br><br>Energy Composition<br>Fat: ~40% (mainly PUFA and MUFA)<br>Carbohydrates: <40g/day in 1st 2 months, then increased up to 80g/day.<br>Less/avoid red + processed meats.<br>Polyphenols: 1240mg/day (28g/day walnuts, 3-4 cups/day green tea, 100g/day Wolffia globosa green shake for dinner)<br>Reduced Poultry intake

					sugars, salt and Increase intake of vegetables	Dietary guidance: Limit dietary cholesterol, trans-fat, saturated-fat, sugars, salt and Increase intake of vegetables.
**LCD vs LCarbD**						
Browning, 2011<br>[32]	USA<br>RCT	LCD vs LCarbD<br><br>Arm 1: LCarbD<br>Arm 2: LCD	University of Texas Southwestern Medical Centre	**LCarbD**<br><br>Teaching session with dietician<br>7 days of detailed food diary then 7 days of frozen meals prepared by research kitchen<br><br>Dietary Composition<br>Carbohydrates: <20g/day	**LCD**<br><br>Teaching session with dietician<br>14 days of prepared meals by research.<br><br>Dietary composition<br>Kcal: ~1200kcal/day women, 1500kcal/day males	
**VLCKD**						
Cunha, 2020<br>[33]	Brazil<br>RCT<br>12/2016 – 05/2018	VLCKD vs LCD<br><br>Arm 1: LCD<br>Arm 2: VLKCD	Patients referred for weight loss treatment clinic of the Intituto Estadual de Diabetes e Endocrinologia, Rio De Janeiro, Brazil	**LCD**<br><br>1400-1800kcal/day<br><br>Dietary Composition<br>Carbohydrates: 45-55%<br>Proteins: 15-25%<br>Fat: 25-35%<br>Fibre:20-40gr/d (vegetables/fruit)	**VLKCD**<br><br>600-800kcal/day<br>Ketogenic diet = VLCD (600-800kcal/day), low carbohydrates (<50gr/day from vegetables), lipids (10gr/day olive oil)<br><br>Dietary Composition<br>Carbohydrates: <50gr/d<br>Lipid: 10gr/d olive oil<br>Protein: 0.8-1.2gr/kg of body weight per day.<br>Vitamins (Vitamin A, 600 mcg; Vitamin D, 5.0 mcg; Vitamin E, 10mg; Vitamin B1, 1.2 mg; Vitamin B2, 1.3mg; Vitamin B6, 1.3mg; Vitamin C, 45 mg; Vitamin B5, 5.0mg; Nicotinamide, 16mg; Folic acid, 240 mcg; Biotin 30 mcg; Vitamin B12, 2.4 mcg; Vitamin K, 65 mcg; Iron, 14mg; Iodine, 130 mcg; Manganese, 2.3mg; Selenium, 34 mcg and trace elements (Sodium: 1g/d, Potassium:1.6 g/d, Calcium: 800 mg/d; Magnesium: 375 mg/d))	
**LCD MUF vs LCD PUF**						

Aller, 2014 [35]	Spain<br>RCT	LCD-MUF vs LCD-PUF<br><br>Arm 1: LCD-MUF (mono-unsaturated fat)<br>Arm 2: LCD-PUF (polyunsaturated fat)	Not Stated	**LCD (enriched with monounsaturated fat)**<br><br>1342kcal/day<br>180min/week aerobic exercise (60min each)<br><br>Energy Composition<br>Carbohydrates: 46.6%<br>Lipids: 34.1%<br>Proteins: 19.2%<br><br>Distribution of Fats<br>Saturated Fat: 21.7%<br>Monounsaturated Fat: 67.5%<br>Polyunsaturated Fat: 10.8%	**LCD (enriched with polyunsaturated fats)**<br><br>1459kcal/day<br>180min/week aerobic exercise (60min each)<br><br>Energy Composition<br>Carbohydrates: 45.7%<br>Lipids: 34.4%<br>Proteins: 19.9%<br><br>Distribution of Fat<br>Saturated Fat: 21.8%<br>Monounsaturated Fat: 55.5%<br>Polyunsaturated Fat: 22.7%

Reference: [12,13,22,26–40]

Table S2: Demographic characteristics of the Included Studies

Intervention Type	Study	Sample Size (n)	Duration	Age (years)	Sex	Ethnicity	Adherence	NAFLD %	Diabetes %	Metabolic Syndrome %	BMI / Body Composition
LCD vs Control	Asghari, 2022 [41]	T: 60<br>C: 30<br>LCD: 30	12w	T: 39.7 ± 6.3<br>C: 39.27 ± 5.51<br>LCD: 40.08 ± 7.08	21 F<br>39 M<br><br>65.0% M	Middle Eastern	83.3%<br>4 FTFU<br>6 non-compliance	100%	Not stated	Not stated	C: 30.41 ± 3.39<br>LCD: 31.32 ± 3.31
	2020, Chen [36]	Total: 44<br>C: 22<br>I: 22	2m	T: 38.1±9.4	16F<br>28M<br>63.6% M	East Asian	-	100%	Not Stated	Not Stated	All participants BMI >= 25
	Elias, 2010 [37]	T: 31<br>Adherent: 17<br>Non-adherent: 14	6m	T: 47.5 ± 11.6<br>Adherent: 47.6 ± 12.9<br>Non-adherent: 47.4 ± 10	16 F<br>15M<br><br>48.3% Male	Hispanic and Latino	NA	100%	Not Stated	45%	Adherent: 32.8 ± 4<br>Non-adherent: 34.5 ± 5.4
	El-Kader, 2016 [38]	T: 100<br>C: 50<br>LCD: 50	3m	T: 51.0 ± 5.6<br>C: 51.12 ± 5.86<br>LCD: 50.78 ± 5.34	30 F<br>70 M<br><br>70%M	Middle Eastern	Not stated	100% NASH	Not Stated	Not Stated	C: 31.76 ± 2.92<br>LCD: 32.35 ± 2.54
	Promrat, 2009 [39]	T: 31<br>C: 10<br>Lifestyle: 21	11m	T: 48.5 ± 11.3<br>C: 47.6 ± 12.0<br>Lifestyle: 48.9 ± 10.9	9 F<br>22 M<br><br>70.9% M	84% Caucasian	90.2%<br>3 drop outs lifestyle group	100% NASH	48% T2D	74%	C: 33.7 ± 4.7<br>Lifestyle: 33.6 ± 5.3

TMR vs Control	Taylor, 2018 [12,13]	T: 90<br>C: 26<br>I: 64	12m	T: 52.8 ± 7.9	38F<br>52M<br><br>57.8% M	98.3% Caucasian	78.5%<br>32/149 TMR group withdrew	Not stated	100%<br>Mean duration: 3.0 ± 1.7 years	Not stated	C: 34.2 ± 4.3<br>LCD: 35.1 ± 4.5<br>T: 34.7 ± 7.4
	Sattar, 2022 [40]	T: 25<br>C: 12<br>I: 13<br><br>Observational: 25	3m	T: 45.8 ± 11.1	12 F<br>13 M<br>52% M	South Asian	84%<br><br>4/25 withdrew during RCT<br>2 Further withdrew during delayed TDR	Not Stated	100%: mean duration since diagnosis 1.9 years	Not stated	T: 32.1 ± 3.8<br>TDR: 32.6 ± 4.4<br>C: 31.6 ± 3.3
MED LCD vs Control	CENTRAL<br>Gepner, 2019 [28,29]	T: 278<br>LFD: 139<br>MED: 139	18m	T: 47.9 ± 9.3	31 F<br>247 M<br>89% M	Middle Eastern - Israel	86.3%<br>LFD: 118<br>MED: 122	53%	11%	40%	30.8 ± 3.8
	FLiO<br>Marin-Alejandre, 2019/2022 (Nutrients / Liver International) [30,31]	T: 98<br>AHA: 48<br>FLiO: 50	6-24m	T: 50.1 ± 9.3<br>AHA: 51.1 ± 9.8<br>FLiO: 49.2 ± 8.9	47 F<br>51 M<br><br>52% M	Caucasian	6m: 77.6%<br>AHA 37, FLiO 39<br>24m: 60.4%<br>AHA 32, FLiO 26	100%	12.2%	Not Stated	AHA: 33.7 ± 4<br>FliO: 33.3 ± 4
	Montemayor, 2022 [26]	T: 128<br>CD: 43<br>MD-HMF: 43<br>MD-PA: 42	12m	T: 52.9 ± 7.27<br>CD: 54.1 ± 8.9<br>MD-HMF: 52.3 ± 7.1<br>MD-PA: 52.2 ± 5.8	47 F<br>81 M<br><br>63.3% M	Caucasian	Not stated	100%	24.2%	100%	CD: 33.6 ± 3.7<br>MD-HMF: 33.9 ± 3.9<br>MD-PA: 33.4 ± 3.1
	Ristic-Medic, 2020 [34]	T: 27<br>LFD: 13<br>MED: 14	3m	T: 33.6 ± 4.2<br>LFD: 34.42 ± 4.66<br>MED: 32.92 ± 3.78	0 F<br>27 M<br><br>100% M	Caucasian	88.9%<br>LFD: 12<br>MED: 12	100%	0%	Not Stated	LFD: 30.17 ± 2.28<br>MED: 30.43 ± 1.81

Green-MED	DIRECT-Plus Meir, 2021 [22,27]	T: 294<br>C: 98<br>MED: 98<br>Green-MED: 98	18m	T: 51.1 ± 10.5	35 F<br>259 M<br>88.1% M	Middle Eastern - Israel	89.8% at 18 months<br>C: 91<br>MED: 84<br>Green-MED: 89	62%	10.9%	Not Stated	31.3 ± 4<br><br>58.8% with obesity
LCarbD	Browning, 2011 [32]	T: 18<br>LCarbD: 9<br>LCD: 9	2w	T: 44.5 ± 11.5<br>LCarbD: 42 ± 11<br>LCD: 47 ± 12	13F<br>5M<br><br>27.8% Male		100%	100%	Not Stated	Not Stated	LCardD: 36 ± 4<br>LCD: 34 ± 9
VLCKD	Cunha, 2020 [33]	T: 46<br>LCD: 24<br>VLCKD: 22	2m	T: 40.3 ± 11.3	38F<br>8M<br><br>17.4% M	Hispanic and Latino	84.8%<br>LCD: 19<br>VLCKD: 20	92.3%	0%	Not Stated	35.7 ± 4.3
LCD MUF vs PUD	Aller, 2014 [35]	T: 44<br>Diet M: 26<br>Diet P: 18	3m	T: 49.3 ± 16.7	32 F<br>12 M<br><br>28.1% M	Caucasian	Not stated	100%	0%	Not stated	Diet M: 39.4 ± 5.4<br>Diet P: 38.9 ± 5.3

Reference: [12,13,22,26–40]

Table S3: Studies Nearly meeting Inclusion

Study	Intervention	Reason exclusion
Zade et al, 2016, Liver International [1]	RCT<br>DASH Diet	Calorie reduction did not meet LCD definition
Luukkonen et al, 2020, PNAS [2]	Low-calorie Ketogenic Diet	No control group
Ryan et al, 2007, Diabetes Care [3]	RCT post-hoc analysis<br>LCD either 60% carb/25% fat or 40% carb/45% fat	Does not define NAFLD prevalence.
Scragg, Clinical and Translational Gastroenterology, 2020 [4]	VLCD	No control group
CALERIE, Larson- Meyer, Obesity, 2008 [5]	RCT<br>LCD or Control	No NAFLD participants
Guo, Journal of Obesity, 2018 [6]	RCT<br>LCD vs Control	No NAFLD participants
Shirai, Obesity Research & Clinical Practice, 2013 [7]	RCT<br>LCD with partial use of formula diet or conventional LCD	No NAFLD participants
DIADEM-I, Taheri, 2020 [8]	RCT<br>TMR vs control	No NAFLD participants
DROPLET, BMJ [9]	RCT<br>TMR	Did not define NAFLD prevalence

References:

[1] Razavi Zade M, Telkabadi MH, Bahmani F, Salehi B, Farshbaf S, Asemi Z. The effects of DASH diet on weight loss and metabolic status in adults with non-alcoholic fatty liver disease: A randomized clinical trial. Liver Int 2016;36:563–71. https://doi.org/10.1111/liv.12990.

[2] Luukkonen PK, Dufour S, Lyu K, Zhang XM, Hakkarainen A, Lehtimäki TE, et al. Effect of a ketogenic diet on hepatic steatosis and hepatic mitochondrial metabolism in nonalcoholic fatty liver disease. Proc Natl Acad Sci U S A 2020;117:7347–54. https://doi.org/10.1073/PNAS.1922344117.

[3] Ryan MC, Abbasi F, Lamendola C, Carter S, Tracey Lynn Mclaughlin. Fat Restriction in Insulin-Resistant Adults. Diabetes Care 2007;30:1075–80. https://doi.org/10.2337/dc06-2169.Abbreviations.

[4] Scragg J, Avery L, Cassidy S, Taylor G, Haigh L, Boyle M, et al. Feasibility of a Very Low Calorie Diet to Achieve a Sustainable 10% Weight Loss in Patients With Nonalcoholic Fatty Liver Disease. Clin Transl Gastroenterol 2020;11:e00231. https://doi.org/10.14309/ctg.0000000000000231.
[5] Larson-Meyer DE, Newcomer BR, Heilbronn LK, Volaufova J, Smith SR, Alfonso AJ, et al. Effect of 6-month calorie restriction and exercise on serum and liver lipids and markers of liver function. Obesity 2008;16:1355–62. https://doi.org/10.1038/oby.2008.201.
[6] Guo X, Xu Y, He H, Cai H, Zhang J, Li Y, et al. Effects of a Meal Replacement on Body Composition and Metabolic Parameters among Subjects with Overweight or Obesity. J Obes 2018;2018. https://doi.org/10.1155/2018/2837367.
[7] Shirai K, Saiki A, Oikawa S, Teramoto T, Yamada N, Ishibashi S, et al. The effects of partial use of formula diet on weight reduction and metabolic variables in obese type 2 diabetic patients - Multicenter trial. Obes Res Clin Pract 2013;7:e43–54. https://doi.org/10.1016/j.orcp.2012.03.002.
[8] Taheri S, Zaghloul H, Chagoury O, Elhadad S, Ahmed SH, El Khatib N, et al. Effect of intensive lifestyle intervention on bodyweight and glycaemia in early type 2 diabetes (DIADEM-I): an open-label, parallel-group, randomised controlled trial. Lancet Diabetes Endocrinol 2020;8:477–89. https://doi.org/10.1016/S2213-8587(20)30117-0.
[9] Astbury NM, Aveyard P, Nickless A, Hood K, Corfield K, Lowe R, et al. Doctor Referral of Overweight People to Low Energy total diet replacement Treatment (DROPLET): Pragmatic randomised controlled trial. BMJ 2018;362. https://doi.org/10.1136/bmj.k3760.

Supplementary Results S1

3.7 Other LCD Interventions

3.71 Very Low Calorie Ketogenic Diet An open-label RCT assessed the very-low calorie ketogenic diet (VLCKD) vs LCD. The trial recruited 39 participants (20 = VLCKD, 19 LCD) to a 2 month intervention. The VLCKD intervention involved a 2-month ketogenic phase with 600-800kcal/day, low carbohydrate intake (<50gr/day), this was based on a commercial weight loss programme (Pronokal method) The participants were 40.3 ± 11.3 years old, predominantly female (82.6% F, 17.4% M), 92.3% were with NAFLD, and average BMI at baseline was 35.7 ± 4.3kg/m2. Two authors in the study author list were personnel of Pronokal Group Brasil, and were involved in the study design and revision of the manuscript, potentially highlighting a conflict of interest. The corresponding author also works for the Pronokal group. This trial showed that ALT, AST and GGT did not significantly differ between groups. Percentage Fat fraction via MRI PDFF reduced to a greater extent with VLCKD (-4.77 ± 4.26%) than LCD (-0.79 ± 1.76) (p = 0.0006 absolute reduction between groups). There were improvements in NAFLD prevalence in the VLCKD group compared to LCD (p = 0.0351). BMI and Body weight reduced to a greater extent in the VLCKD (VLCKD: -9.7 ± 3.88kg, LCD: -1.67 ± 2.22kg, $p < 0.0001$) [41].

3.72 Low-Calorie Diet vs Low-Carbohydrate Diet Browning et al investigated LCD vs Low Carbohydrate Diet (LCarbD). The trial was two weeks duration and recruited 18 participants with NAFLD and obesity [52]. This trial was in the USAs, most participants were female (13F, 5 M). Calories reduced more significantly in LCD, but this was not significant. LCarbD had 8% energy from carbs, LCD was 50% from carbs. LCarbD had 33% energy from protein whereas LCD was 16%. LCarbD was 59% whereas LCD was 34%. Following two weeks of intervention ALT, AST and AST:ALT ratio change did not significantly vary between groups. MR-Spectroscopy determined liver fat significantly reduced in LCarbD group vs LCD (p = 0.049). BMI and body weight did not significantly differ between groups in terms of change.

3.73 High monounsatured Fat LCD (LCD-MUF) vs High polyunsatured Fat LCD (LCD-PUF) Aller et al conducted a RCT in Spain evaluated high monounsatured fat LCD vs high polyunsatured fat LCD in participants with NAFLD [32]. Both groups underwent a calorie restricted diet with 180min/week aerobic exercise. LCD-PUF was high in polyunsatured fat; LCD-MUF was higher in monounsatured fat. Intervention was 3 months, 44 participants were enrolled (LCD-mono 26, LCD-poly 18), average age was 49.3 ± 16.7, most participants were female (28.1%). All participants had NAFLD, no participants had diabetes. Attrition rate and prevalence of metabolic syndrome not reported. Average BMI at baseline was 38.9 – 39.4. Between group statistics were not reported. ALT, AST and GGT significantly reduced in both groups. BMI, Body weight and Body Fat % significantly reduced in both groups.
